# The All Babies Count Initiative: Impact of a Health System Improvement Approach on Neonatal Care and Outcomes in Rwanda

**DOI:** 10.9745/GHSP-D-20-00031

**Published:** 2020-09-30

**Authors:** Hema Magge, Evrard Nahimana, Jean Claude Mugunga, Fulgence Nkikabahizi, Elisabeth Tadiri, Felix Sayinzoga, Anatole Manzi, Merab Nyishime, Francois Biziyaremye, Hari Iyer, Bethany Hedt-Gauthier, Lisa R. Hirschhorn

**Affiliations:** a Division of Global Health Equity, Brigham and Women’s Hospital, Boston, MA, USA.; b Division of General Pediatrics, Boston Children’s Hospital, Boston, MA, USA.; c Partners In Health/Inshuti Mu Buzima, Rwinkwavu, Rwanda.; d Partners In Health, Boston, MA, USA.; e Ministry of Health, Government of Rwanda, Kigali, Rwanda.; f Center for Global Health, Massachusetts General Hospital, Boston, MA, USA.; g Department of Epidemiology, Harvard T.H. Chan School of Public Health, Boston, MA, USA.; h Department of Social Medicine, Harvard Medical School, Boston, MA, USA.; i Department of Medical Social Sciences, Northwestern University Feinberg School of Medicine, Chicago, IL, USA.

## Abstract

**Introduction:**

Poor-quality care contributes to a significant portion of neonatal deaths globally. The All Babies Count (ABC) initiative was an 18-month district-wide approach designed to improve clinical and system performance across 2 rural Rwandan districts.

**Methods:**

This pre-post intervention study measured change in maternal and newborn health (MNH) quality of care and neonatal mortality. Data from the facility and community health management information system and newly introduced indicators were extracted from facility registers. Medians and interquartile ranges were calculated for the health facility to assess changes over time, and a mixed-effects logistic regression model was created for neonatal mortality. A difference-in-differences analysis was conducted to compare the change in district neonatal mortality with the rest of rural Rwanda.

**Results:**

Improvements were seen in multiple measures of facility readiness and MNH quality of care, including antenatal care coverage, preterm labor management, and postnatal care quality. District hospital case fatality decreased, with a statistically significant reduction in district neonatal mortality (odds ratio [OR]=0.54; 95% confidence interval [CI]=0.36, 0.83) and among preterm/low birth weight neonates (OR=0.47; 95% CI=0.25, 0.90). Neonatal mortality was reduced from 30.1 to 19.6 deaths/1,000 live births in the intervention districts and remained relatively stable in the rest of rural Rwanda (difference in differences −12.9).

**Conclusion:**

The ABC initiative contributed to improved MNH quality of care and outcomes in rural Rwanda. A combined clinical and health system improvement approach could be an effective strategy to improve quality and reduce neonatal mortality.

## Abstract (precis)

A health system improvement program combining facility readiness support, clinical training/mentoring, and improvement collaboratives increased quality improvement capacity, improved maternal and newborn quality of care, and reduced neonatal mortality. These results can be used to inform system improvement approach design to transform quality of care and outcomes for newborns.

## Teaser

**Key Findings:**

- A district-wide health system improvement program combining facility readiness support, clinical training/mentoring, and district-wide improvement collaboratives increased quality improvement capacity, improved maternal and newborn quality of care, and reduced neonatal mortality by approximately 35% overall and 49% among high-risk preterm/low birth weight infants.
- This improvement in mortality was not seen during the same time period in the rest of rural Rwanda.

**Key Implications:**

- Policy makers should consider adopting components of the All Babies Count program into the design of system improvement approaches to transform quality of care and outcomes for newborns.
- Embedding the design into existing health system structures could help structure improvement in other clinical domains.

## INTRODUCTION

Significant progress has been made worldwide in under-5 mortality, with more than 50% reduction from 1990 to 2015.^1^ However, the rate of progress in the neonatal period has been slower, with death in the first month of life accounting for 46% of under-5 deaths globally.^2^ In response, a global movement has grown to accelerate progress,^3^ and efforts have prioritized improving access along antenatal, delivery, and postnatal care.^4^^,^^5^ However, interventions targeting access without addressing quality have not resulted in reduction of neonatal mortality^6^; improving care quality could prevent up to an estimate 71% of newborn deaths.^7^

After the health system devastation from the 1994 genocide, Rwanda rebuilt the health sector, experienced a 63% reduction in premature mortality between 2000 and 2011,^8^ and achieved Millenni-um Development Goals 4 and 5. However, similar to the global experience, neonatal mortality reduction lagged behind the success in reducing deaths after the first month of life.^9^ In response, Rwanda prioritized addressing neonatal mortality.^10^ By 2010, Rwanda had tremendous increases in facility-based maternal and newborn health (MNH) care including skilled delivery; therefore, improving facility newborn care quality became a key priority.^9^

Since 2005, Partners In Health (PIH), a global nongovernmental organization with a mission to provide high-quality health services in disadvantaged communities, has supported the Rwanda Ministry of Health (MOH) in district health system strengthening.^11^ In response to the prioritization of reducing neonatal mortality nationally, PIH partnered with MOH to design the All Babies Count (ABC) initiative to improve district-wide quality of newborn care. The objective of this study was to evaluate the impact of ABC on quality improvement (QI) activities, neonatal quality of care, and neonatal mortality across the prenatal, perinatal, and postnatal risk periods in 2 rural Rwandan districts.

This study evaluated the impact of ABC on QI activities, neonatal quality of care, and neonatal mortality across the prenatal, perinatal, and postnatal risk periods.

## METHODS

### Setting

This study was conducted in southern Kayonza (SK) and Kirehe districts in eastern Rwanda, where PIH began support in 2005. The MOH chose these districts in part due to their poor health status post-genocide. The intensity of PIH support was gradually reduced as the 2 districts caught up with the rest of the country in infrastructure, economy, and health status. By 2012, PIH’s partnership had moved from direct care provision and management support toward targeted financial and technical assistance to support clinical innovation areas, including newborn health.^12^

ABC was introduced in 2013 in SK and Kirehe districts, with a population of approximately 500,000 people. At initiation, each district had 1 hospital, with 8 health centers providing maternity services in SK and 13 in Kirehe. These government facilities were administered and funded publicly and received additional technical/financial support for general operations and specialized clinical services through their partnership with PIH. Three additional health centers initiated maternity services in Kirehe during ABC implementation and were integrated into the program. District hospitals were staffed per national standards by general practitioners, nurses, and midwives, and they had the capacity to perform cesarean deliveries. Each district hospital had a neonatal unit as per the national standards to provide care for ill and preterm infants using the national neonatal care protocol including continuous positive airway pressure management.^10^ A US-trained pediatrician supported by PIH was present 6 months per year as part of general hospital support across the 2 districts. Health centers were staffed by nurses and midwives performing routine MNH and under-5 care.

### Program Design

Building upon a mentoring-based QI program that was successful in addressing quality gaps in pediatric care across the 2 districts,^13^ ABC was designed to reduce neonatal mortality by improving the quality of antenatal, delivery, and postnatal care through combining facility-focused clinical and QI mentorship with district-wide QI collaboratives adapted from the Breakthrough Series model (Figure).^14^ Our aim was to ensure facility readiness for quality newborn care at the start, followed by the introduction of a district-wide QI approach to address key mortality drivers along the continuum of antenatal, delivery management, and postnatal care and at all levels of the system. The approach was designed in collaboration with the MOH Maternal and Child Health Department, with feedback from key national and district stakeholders.^15^

**FIGURE.**
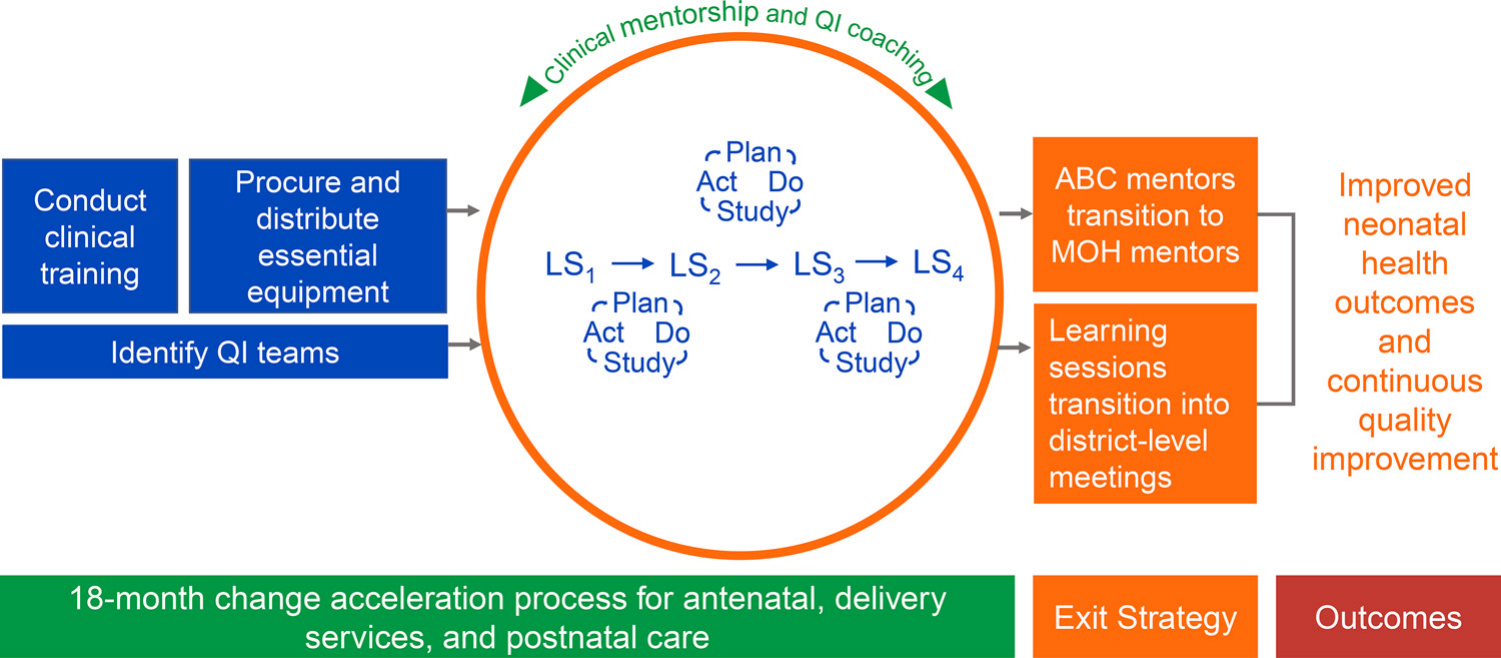
All Babies Count Initiative Design Implemented in 2 Districts in Rwanda Abbreviations: ABC, All Babies Count; LS, learning session; MOH, Ministry of Health; QI, quality improvement.

A baseline quality assessment was completed by the PIH monitoring and evaluation team as part of a routine quarterly health facility survey. By design, the results were used by the ABC team to identify and close gaps in relevant neonatal training and commodities before the first learning session (LS). Gaps were addressed through baseline provision of essential equipment to meet facility national standards and annual relevant clinical trainings to address staff turnover. The QI collaboratives aimed to strengthen health care worker QI and clinical capacity through mentorship; efficiently test a large number of locally designed system interventions, called “change ideas,” to create relevant neonatal change packages; and spread successful changes through LSs.^16^ Each collaborative was based on district leadership and interdisciplinary QI teams from all district facilities (hospital and health centers), including facility directors, midwives, antenatal/postnatal care providers, data officers, and community health supervisors. Hospital QI teams included the clinical director and/or head of maternity, neonatal, and operating theaters. On average, 3–5 team members participated in each LS (total 30–50 participants).

PIH-employed ABC nurse mentors were neonatal clinical experts trained in QI, data quality, and mentorship. They provided the integrated clinical and QI mentorship and supported QI projects to all district facilities (hospital and health centers). They worked alongside district hospital supervisors—whose existing responsibilities included supervising clinical care at health centers—to build their technical and mentorship skills to transition program ownership by the end of the collaborative. ABC mentors and district hospital supervisors worked together on core program implementation elements including data collection, facility mentorship, and LS organization and facilitation.

### Study Design

The program evaluation was a pre-post intervention design measuring change in MNH quality of care and neonatal mortality. Embedded into the study were measurements of program implementation, QI activities, facility readiness, and patient satisfaction. Stillbirths were tracked as a secondary outcome. Baseline was July–September 2013 with the first LSs in October 2013. Because neonatal mortality had seasonal variability, the endpoint quarter was chosen 3 months after the last LS to capture the same season (July–September 2015). Indicators that were newly introduced had a baseline of October–December 2013 (Table 1).

**TABLE 1.** Selection of Maternal and Newborn Health Core Improvement Collaborative Indicators and Outcome Measures Used in Evaluating an All Babies Count Initiative Implemented in Rwanda

	Indicator	Numerator	Denominator	Data Source	Facility
Antenatal care^a^	1. ANC standard 4 visit coverage	Number of women who delivered in a HF who had 4 ANC visits	Total number of health facility deliveries PLUS total number of referrals of laboring women from HC to hospital PLUS home deliveries	HMIS	HCs
Delivery services	2. Percentage of pregnant women with facility delivery	Total number of health facility deliveries	Total number of home deliveries PLUS total number of health facility deliveries	Community HMIS	HCs
	3. Time to cesarean delivery for emergency (from determination of need at hospital to time of delivery)	Average time to cesarean delivery for emergency (from determination of need by doctor at hospital to time of cesarean delivery incision)		Chart review^b^	Hospital
	4. Antenatal steroids for preterm labor	Number of women with preterm labor <34 weeks treated with dexamethasone	Number of women with preterm labor (<34 weeks)	Chart review	Hospital (HCs added later)
Postnatal care	5. Percentage of babies with immediate skin-to-skin after delivery	Number of babies placed immediately skin to skin	Number of babies born vaginally	Chart review	HCs and hospital
	6. Danger sign assessment within 24 hours	Newborns checked for danger signs in postpartum ward within 24 hours	Total deliveries MINUS stillbirths macerated MINUS stillbirths fresh MINUS death at birth of live born babies	HMIS	HC and hospitals
Outcome	7. Neonatal unit case fatality	Number of deaths in neonatal unit (<28 days)	Number of admissions to neonatal unit (<28 days)	Chart review	Hospital
	8. District-wide neonatal mortality	HMIS neonatal deaths PLUS death at birth of live born babies PLUS number of neonatal deaths at community	Live births PLUS number of home deliveries	HMIS	HCs and hospital
	9. Facility neonatal mortality among preterm/LBW^c^	Hospital deaths due to prematurity PLUS HC deaths due to prematurity	Hospital LBW/non-preterm PLUS hospital preterm PLUS HC LBW/non-preterm PLUS HC preterm	HMIS	HCs and hospital
	10. Facility neonatal mortality among non-preterm/LBW^d^	Hospital deaths of all causes except prematurity PLUS HC deaths of all causes except prematurity	Hospital and HC live births MINUS (hospital LBW/non-preterm PLUS hospital preterm PLUS HC LBW/non-preterm PLUS HC preterm)	HMIS	HCs and hospitals
	11. Facility stillbirths (macerated and fresh)^c^	Stillbirths macerated PLUS stillbirths fresh	Total deliveries	HMIS	HCs and hospital

Abbreviations: ANC, antenatal care; HC, health center; HF, health facility; HMIS, health management information system; LBW, low birth weight.

a Rwinkwavu health center did not have delivery services, so the denominator was changed to number of community HMIS health facility deliveries at district hospital plus number of community HMIS home deliveries.

b Chart review indicators were newly introduced at the start of the intervention.

c Prematurity defined as gestational age ≤37 weeks, and low birth weight defined as birth weight < 2500 g per World Health Organization standard definition.

d Stillbirths defined in national HMIS data dictionary as a baby born with no signs of life at or after 22 weeks gestation and with birth weight greater than or equal to 500 g. Stillbirth analysis restricted to facility level given the absence of community-based recording of stillbirths.

### Measures

#### Program Implementation

We measured the implementation process and outcomes including domains of feasibility and fidelity based on the framework published by Proctor et al.^17^ The data collected included trainings and equipment provided, mentoring frequency and content, LS attendance, and change ideas tested by facilities. These data were used to strengthen program delivery and allow for adaptations to maximize impact. Program dose was monitored so changes could be detected and responded to promptly to maintain implementation fidelity.

#### QI Activities and Facility Readiness

The measurement of facility QI activities included the number of projects executed and change ideas tested. Facility readiness was based on national facility neonatal standards including minimum staffing, equipment, and medications (Supplement 1).^18^

#### MNH Quality of Care and Neonatal Mortality

MNH quality indicators were selected based on literature review, MOH priorities, and existing health management information system (HMIS) data to represent the evidence-based care pathways: antenatal, delivery management, and postnatal care (Table 1). Measurement of asphyxia and measurement of provision of antibiotics for premature preterm rupture of membranes were introduced, but reliable capture was found to be a challenge for providers due to difficulty with systematic documentation. Despite being unable to reliably track the number of newborns with asphyxia, asphyxia prevention and management was a core focus of clinical mentorship and ad-dressed through many QI change ideas targeting an aspect of delivery management. Hospital neonatal unit and district-wide neonatal mortality was measured using all available data (including births and deaths in the community). Mortality among preterm/low birth weight (LBW) infants was estimated using available data (facility only) as a particularly high-risk subpopulation and a leading cause of neonatal mortality.

### Data Collection

Implementation data and QI activities were extracted from routine program tools. Measurement of essential equipment, medications, and training standards were assessed quarterly using standardized service readiness surveys.

MNH process and mortality data from facility HMIS were extracted and compared with paper registers as part of QI coaching. Data for new indicators were collected directly from additional facility registers and through weekly random sampling of charts for cesarean delivery time. Births and deaths in the community were collected from community HMIS and compared with community health worker supervision records from the corresponding facilities. District neonatal mortality for the rest of rural Rwanda was constructed from the HMIS as register review was not feasible.

Patient satisfaction data were collected using surveys measuring experience of care and satisfaction on a Likert scale from 1 (poor) to 5 (excellent) of a sample of women attending antenatal care (ANC) and delivery services in all intervention facilities. This study included 278 women from Kirehe (ANC 204, maternity 74) and 198 from SK (ANC 166, maternity 32) with baseline collection from November to December 2013, and endpoint collection from July to September 2015. Detailed methods are described elsewhere.^19^

Patient satisfaction data were collected by surveys completed by a sample of women attending ANC and delivery services in all intervention facilities.

### Analysis

#### Program Implementation, MNH Quality of Care, and Neonatal Unit Case Fatality

We constructed the numerator, and where appropriate the denominator, by calculating the mean monthly value for the 3-month period corresponding to the baseline or endpoint quarter. For each time period, we report the median of the numerator and the median of the denominator. For indicators 1–4, 6, 8, and 9 in Table 2, we report the median and interquartile range for the health facility and assessed changes using a Wilcoxon signed rank test at the α=.05 significance level. Analyses were conducted in Stata SE v14 (College Station, TX).

**TABLE 2.** Change in Maternal and Newborn Health Quality of Care Indicators in All Babies Count Initiative Implemented in 2 Districts in Rwanda

Indicator	Aggregate				South Kayonza			Kirehe		
	Baseline	Endpoint	*P* Value	Median Difference (IQR)	Baseline	Endpoint	*P* Value	Baseline	Endpoint	*P* Value
	Median (IQR)	Median (IQR)			Median (IQR)	Median (IQR)		Median (IQR)	Median (IQR)	
1. Percentage of deliveries where mothers had 4 standard ANC visits	13.7 (6.7, 44.2)	30.4 (15.1, 43.1)	.04	13.6 (−3.3, 26.4)	42.4 (19.9, 47.3)	36.4 (13.5, 66.3)	.78	10.2 (2.8–26.8)	29.6 (18–34.2)	.02
2. Percentage of pregnant women delivering in facilities	89.6 (86.3, 94.9)	92.6 (86.8, 95.8)	.25	7.8 (−2.8, 5.1)	92.2 (87.2, 95.3)	95.2 (93.0, 96.3)	.33	88.4 (86.3–96.7)	91.0 (86.2–94.6)	.36
3. Percentage of babies who are provided immediate skin-to-skin after birth	53.6 (0, 80.9)	97.4 (96.4, 99.3)	<.001	43.6 (17.7, 95.7)	91.2 (75.9, 100)	99.6 (96.7, 100)	.18	4.3 (0, 53.6)	97.2 (96.1, 98.8)	<.001
4. Percentage of newborns checked for danger signs within 24 hours of birth	46.6 (31.1, 96.7)	98.7 (96.4, 100)	<.001	47.7 (−1.4, 67.1)	52.3 (33.8, 98.6)	100 (93.8, 100)	.06	45.7 (15.8, 82.1)	98.6 (97.4, 98.8)	.003
5. Average hospital time to emergency cesarean delivery (minutes)					167	50	—	82	61	—
6. Percentage of women with preterm labor who are treated with antenatal steroids	0 (0, 0)	41.7 (0, 100)	.32	—	0 (0, 0)	16.7 (0, 33.3)	—	0 (0–0)	75 (0, 100)	.32
7. Percentage of facilities with at least 2 MNH clinically trained staff	100	100	—	15.1 (4, 29.8)	100	100	—	100	100	—
8. Percent availability of essential medications for MNH care	61.2 (45.0, 77.8)	81.8 (72.7, 81.8)	<.001	31.2 (19.5, 37.8)	35.0 (25.0, 45.0)	83.3 (75.0, 87.5)	.01	66.7 (55.6, 77.8)	77.8 (72.7, 81.8)	.05
9. Percent availability of functioning equipment essential for MNH care	55.6 (48.2, 61.1)	86.6 (77.8, 88.9)	<.001	—	55.6 (47.2, 56.6)	88.9 (88.9, 94.4)	.01	55.6 (48.2, 63.9)	81.1 (77.8, 88.6)	<.001
10. Patient satisfaction^a^: average satisfaction with ANC	—	—	—	—	2.8 (SD: 1.57)	3.1 (SD: 1.53)	.11	3.3 (SD: 1.62)	3.6 (SD: 1.52)	.01
11. Patient satisfaction: average satisfaction with maternity care	—	—	—	—	2.4 (SD: 1.67)	2.3 (SD: 1.160	.95	3.5 (SD: 1.59)	3.4 (SD: 1.52)	.61

Abbreviations: ANC, antenatal care; IQR, interquartile range; MNH, maternal and newborn health; SD, standard deviation.

a Patient satisfaction scores on a Likert scale: 1=excellent; 2=very good; 3=good; 4=fair; 5=poor.

#### Patient Satisfaction

We assessed the difference in the proportion reporting high (Likert score=4 or 5) patient satisfaction between baseline and endpoint using a chi-squared test at the α=.05 significance level.

#### Neonatal Mortality and Stillbirths

To estimate neonatal mortality at the level of the individual birth or death rather than as a population estimate aggregated to facility level, we expanded the dataset such that each row corresponded to either a neonatal death or live birth. To offset the power increase obtained by artificially increasing the number of observations, we included a categorical variable for health facility to use as a random intercept and account for clustering. This pseudo-dataset contained the following variables: indicator for death (1=death/0=live birth), time period (1=baseline, 0=post), health facility (categorical for each health facility), and district (1=SK, 0=Kirehe). Based on our hypothesis that mortality could differ based on gestational age/birth weight, mortality estimates were calculated for each subgroup. For each mortality measure, we used mixed-effects logistic regression models to assess changes in mortality with a random effect for health facility to account for clustering (Supplement 2). We tested for interactions between intervention and district with an intervention-district interaction term included in our regression model. If the interaction term was statistically significant, we reported the intervention effect stratified by district. When the interaction term was not significant, we reported a collapsed effect (Supplement 3). The models were fit using SAS v 9.4 and did not control for other co-variates because they were not available on the individual level.

#### Neonatal Mortality Difference in Differences

Change in mortality was defined as the difference of population deaths per 1,000 live births between baseline and endpoint. The difference in differences was examined for the intervention districts against HMIS-reported data from the rest of Rwanda (24 districts with 8.4 million people,^9^ excluding urban Kigali and Burera District). Burera District was excluded because it received PIH neonatal clinical and infrastructure support, but not the complete ABC program. Population district mortality for the national comparison districts included all HMIS mortality data available from the relevant quarters.

### Ethical Considerations

Informed consent was obtained from women surveyed for patient satisfaction.^19^ This study was approved by the Institutional Review Board of Brigham and Women’s Hospital (2009-P-001941/11; BWH) and the Rwanda National Ethics Committee (RNEC 032/RNEC/2012).

### Patient and Public Involvement

The study was supported by a community advisory group composed of separate focus groups with women from the intervention area, community health workers, traditional healers, and facility nurses and doctors. The advisory group provided input for the initial program conceptual framework and intervention design. They were not specifically involved in the evaluation methods design because the evaluation was predominantly conducted using routine programmatic data. Findings from the study have been disseminated with key stakeholders within the health system and communities affected by the work.

### Role of the Funding Source

The funder was not involved in study design, execution, or preparation of this manuscript.

## RESULTS

### Program Implementation and QI Activities

The ABC initiative was implemented in 2 districts and reached all facilities, with full participation by district leadership and the facility QI teams. There was no significant change in clinical staffing of health centers (baseline 9, endpoint 10; *P*=.09). Fidelity include mentoring coverage. Facilities received an average of 0.68 visits/month, and a change package of 46 successful change ideas was developed by the endpoint to facilitate spread.^16^ ABC adaptation informed by quarterly implementation data was undertaken to increase acceptability and adoption. For example, initially LSs were planned quarterly to complete the collaborative within 1 year. However, during LS2, it was found that more time was needed for data quality stabilization, introduction of data sources for new indicators (or HMIS indicators without clear register sources), QI project implementation, and data monitoring and coaching activities. In response, the collaborative duration was adapted to 18 months to extend action periods to accommodate having adequate time for change idea testing. Adoption of ABC components by facilities was seen, including QI (with a median of 2 QI projects running at endpoint, a total of 118 change ideas were tested across the 2 collaboratives), and complete facility attendance at all LSs in both districts.

### Facility Readiness, MNH Quality of Care, and Mortality

Table 2 shows changes in quality indicators that were the focus of QI activities. Facility readiness improved significantly, including availability of essential medications (median difference=15.1%; interquartile range [IQR]=4%, 29.8%; *P*<.001) and equipment (median difference=31.2%; IQR=−19.5%, 37.8%; *P*<.001). As planned, clinical trainings were conducted before the baseline quality of care data collection; all facilities had at least 2 MNH-trained staff, Rwinkwavu district hospital had 8 neonatal intensive care unit-trained staff at baseline and endpoint, and Kirehe increased from 7 to 14. The specific individuals may have changed. Significant improvement in ANC coverage (median difference 13.6%; IQR −3.3%, 26.4%; *P*=.04), provision of immediate skin-to-skin (median difference=43.6%; IQR=17.7%, 95.7%; *P*<.001), and danger signs assessment (median difference=47.7%; IQR=−1.4%, 67.1%; *P*<.001), was seen across the intervention districts. Results in these measures improved across both districts with the exceptions of ANC coverage and immediate skin-to-skin, which had higher baselines in SK. Facility delivery rate had baseline values approaching 90% in both districts, and rates were sustained (median difference=7.8%; IQR=−2.8%, 5.1%; *P*=.25). District-level improvement was seen in complications management: steroid administration for preterm labor increased from 0 to 41.7% (median difference=0%; IQR=0%, 100%; *P*=.32 across both) and time from cesarean delivery decision to incision decreased in SK from 167 to 50 minutes and in Kirehe from 82 to 61 minutes). District hospital neonatal unit case fatality decreased from 28.2% to 12.2% in SK and from 23.4% to 10.1% in Kirehe.

Significant improvement in ANC coverage, provision of immediate skin-to-skin contact, and danger signs assessment was seen.

Patient-reported satisfaction with care for both ANC and maternity services had minimal change. For ANC, no significant change was seen in SK (from 2.8 to 3.1; *P*=.11) and minimal improvement was seen in Kirehe (from 3.3 to 3.6, *P*=.01). No change was seen in either district for satisfaction with maternity care.

Table 3 presents results from the mixed-effects logistic regression models estimating change in neonatal mortality associated with ABC implementation. District neonatal mortality significantly decreased overall from 30.1 to 19.6 deaths/1,000 live births (adjusted odds ratio [aOR]=0.54; 95% confidence interval [CI]=0.36, 0.83). Among preterm/LBW neonates, mortality decreased from 198.8 to 100.6 deaths/1,000 preterm/LBW live births (aOR=0.47; 95% CI=0.25, 0.90). Mortality among non-preterm infants had a nonsignificant decrease from 10.4 to 7.5 deaths/1,000 non-preterm/LBW live births (aOR=0.60; 95% CI=0.36, 1.02). Stillbirths were the only outcome for which district was found to be a significant effect in the model and are reported by district. A nonsignificant decrease was found in Kirehe (OR=0.90; 95% CI=0.61, 1.32), and there was a significant increase in SK (OR=1.71; 95% CI=1.06, 2.75).

**TABLE 3.** Change in Neonatal Mortality Across 2 Districts in Rwanda Where All Babies Count Initiative Was Implemented

Indicator	Baseline	Endpoint	aOR (95% CI)^a^
Neonatal mortality (deaths/1,000 live births)			
Aggregate	30.1 (103/3,426)	19.6 (57/2,902)	0.54 (0.36, 0.83)
Southern Kayonza	35.4 (55/1,553)	18.5 (24/1,295)	
Kirehe	25.6 (48/1,873)	20.5 (33/1,607)	
Facility neonatal deaths in preterm infants/1,000 preterm and LBW live births			
Aggregate	198.8 (32/161)	100.6 (18/179)	0.47 (0.25, 0.90)
Southern Kayonza	290.3 (18/62)	134.3 (9/67)	
Kirehe	141.4 (14/99)	80.4 (9/112)	
Facility neonatal deaths in non-preterm babies/1,000 non-preterm and LBW live births in district			
Aggregate	10.4 (36/3,446)	7.5 (24/3,181)	0.60 (0.36, 1.02)
Southern Kayonza	7.9 (11/1,387)	5.9 (7/1,177)	
Kirehe	12.1 (25/2,059)	8.5 (17/2,004)	
Facility stillborn rate (macerated and fresh)/total per 1,000 births			
Aggregate	23.4 (84/3,590)	28.8 (99/3,436)	
Southern Kayonza	20.7 (29/1,398)	34.5 (44/1,274)	1.71 (1.06, 2.75)
Kirehe	25.1 (55/2,192)	25.4 (55/2,162)	0.90 (0.61, 1.32)

Abbreviations: aOR, adjusted odds ratio; CI, confidence interval; LBW, low birth weight.

a Odds estimates from models stratified by district only provided if test for interaction between district and time in regression was statistically significant at 0.05 level.

District neonatal mortality significantly decreased overall and district hospital neonatal unit fatality decreased.

District-level neonatal mortality was compared with the rest of rural Rwandan districts. Neonatal mortality decreased by 45% in the intervention districts, while remaining relatively stable in the rest of rural Rwanda (Table 4). The difference in differences analysis found a notable difference in the change in the intervention area compared with national secular trends (−13.0). Given the lack of register-reported data in the comparison districts, a sensitivity analysis was conducted comparing the HMIS-reported data from the intervention districts, and similarly found a notable difference in differences of −9.2.

**TABLE 4.** Change in Neonatal Mortality in All Babies Count Intervention Area in 2 Districts Compared With the Rest of Rural Rwanda

	Pre-intervention (Deaths/1,000 Live Births)	Post-intervention (Deaths/1,000 Live Births)	Per 1,000 Change
(1) Southern Kayonza/Kirehe (ABC)	30.1 (103/3,426)	19.6 (57/2,902)	−10.4
Southern Kayonza	35.4 (55/1,553)	18.5 (24/1,295)	
Kirehe	25.6 (48/1,873)	20.5 (33/1,607)	
(2) Southern Kayonza/Kirehe (HMIS)	22.3 (87/3,896)	15.6 (59/3,785)	−6.7
Southern Kayonza	28.7	25.3	
Kirehe	18.3	10.7	
(3) Rural Rwanda comparison districts^a^	13.4 (834/62,382)	15.9 (958/60,225)	2.5
	Difference		
Difference 1 − 3	−13.0		
Difference 2 − 3	−9.2		

Abbreviations: ABC, All Babies Count; HMIS, health management information system.

a Bugesera, Gakenke, Gatsibo, Gicumbi, Gisagara, Huye, Kamonyi, Karongi, Muhanga, Musanze, Ngoma, Ngororero, Nyabihu, Nyagatare, Nyamagabe, Nyamasheke, Nyanza, Nyaruguru, Rubavu, Ruhango, Rulindo, Rusizi, Rutsiro, Rwamagana Districts HMIS reported data.

## DISCUSSION

### Implementation Successes

We found that the successful implementation of a multilevel intervention combining facility readiness, clinical mentoring, and district-wide improvement collaboratives increased QI capacity, improved quality of care, and was temporally associated with reduced neonatal mortality overall and among preterm/LBW infants—a high-risk subpopulation—in the intervention districts.^20^ Maternal and neonatal quality of care in low- and middle-income countries has gained attention as increases in service coverage have not been met with anticipated mortality reduction.^6^ Attention is now drawn to health system improvement as a strategy to improve outcomes.^1^

The multilevel intervention increased QI capacity, improved quality of care, and was temporally associated with reduced neonatal mortality overall and among preterm/LBW infants.

We approached neonatal mortality reduction as a district-wide endeavor—a factor that we believe facilitated impact. We aimed to align facility readiness and care provision with national standards and strengthen quality across all facilities acting as a network of care to serve a catchment population. We leveraged the standard improvement collaborative approach—which typically focuses on facilities through a defined learning network^14^—to create a district-wide learning system to accelerate improvement.

Although improvement collaboratives have gained popularity based on success in high-income settings,^21^ evidence of impact in low- and middle-income countries has been mixed.^22^^,^^23^ However, to our knowledge, evidence has not been published regarding collaboratives in low- and middle-income countries adapted to facilitate a district-wide health system approach. We used LSs to convene key stakeholders, from community representatives to district leaders, to facilitate problem solving across traditional hierarchies. MNH quality gaps that require multilevel solutions, such as infrastructure, referral systems, and data quality, were tackled in improvement projects.^16^

The ABC initiative, as with all QI efforts, required ownership by leaders to achieve impact. We achieved this through intensive and proactive stakeholder engagement. It included partnering with national and district leadership to determine what they believed would be necessary to achieve high-quality MNH care, ensure district hospital capacity to manage neonatal complications, and create functioning district-wide referral systems. Evidence of leadership engagement was seen through the prioritization of addressing newborn health in a coordinated manner. Both districts’ leaders included neonatal quality of care and mortality into their public performance contracts (imihigo) to which they were formally accountable to the government, and they led efforts to improve the culture of data reporting and use. We found the inclusion of MNH goals in the district leadership imihigo to be a way to help engage leaders throughout the duration of program implementation and to jointly solve challenges encountered. We saw reduced discrepancies in the HMIS of neonatal deaths over time, indicating some degree of success of these efforts.^16^^,^^24^

Within the collaboratives, we saw broad QI adoption reflected in activated facility teams with QI capability and a high degree of QI activities across facilities. QI is often found to be focused on microlevel facility improvements, resource intensive, and of questionable impact^1^; however, we found this district-wide system approach feasible and effective.

### Implementation Challenges

Furthermore, the QI work endured despite challenges. Illustrative examples include staff turnover, a data culture of “blame,” and changing population needs. First, staff at health centers moved frequently—as is commonly faced in low- and middle-income countries. Although overall staff number remained stable, changes in individuals required an adaptive process to bring new staff in QI teams up to date. We found the improvement collaborative design, when integrated into routine structures, provided a scaffolding for quickly bringing new staff into facility and district QI efforts and enabling them to be clinically mentored and learn QI methods.

Another challenge encountered early in the program related to leadership support at health centers. Leaders were engaged in stakeholder meetings; however, once QI teams began working to address system problems, some participants reported resistance from their supervisors attributed to lack of familiarity with QI methods. As in many settings, creating a culture of data use requires shifting from norms of blaming individuals, to norms of understanding and improving systems. Therefore, mentors and district supervisors trained district and facility leaders in QI methods and included these leaders in LSs to build their QI capability and data fluency.

Contextual changes in the population and health system also posed a challenge. In Kirehe, given the large population size of some facility catchment areas, 3 new health centers were built during the initiative and had to be incorporated into coaching and LSs. Additionally, the district received a large influx of returning Rwandans from Tanzania in 2013. The improvement collaborative design allowed for natural integration of new facilities into LSs, coaching visits, and QI initiatives, as well as customized support to respond to these changes.

More generally, the multilevel design facilitated system gap identification necessary to provide quality care and the integration of multiple methodologies enabled flexible solutions. District mentors grounded QI coaching and built trust with providers through clinical observation and support.^16^ A qualitative study of participating QI teams found that the nontraditional collaborative components—equipment and clinical support and the combined mentorship approach—were factors related to high impact QI initiatives.^16^ Many improvement programs focus on narrow interventions, without taking a comprehensive view of the mortality drivers of MNH care quality across a health system.^3^^,^^7^ Consistent with other research, the Better Birth Trial—a study of the World Health Organization Safe Childbirth Checklist—found that despite improved care practices at individual facilities, there was no impact on mortality, concluding that a greater system improvement focus could be required.^23^^,^^25^

The multilevel design facilitated system gap identification necessary to provide quality care, and the integration of multiple methodologies enabled flexible solutions.

### Variation in Changes Between Districts

We saw district-level variability in the baseline and change associated with the intervention in some quality measures, consistent with prior study in the region.^15^ SK had more facilities for the population than Kirehe, with a better developed road system to facilitate access. These factors could have contributed to lower baseline levels in Kirehe for service utilization indicators. We saw rapid change in ANC in Kirehe, which had low documented coverage at baseline and greater room for improvement. However, although Kirehe had a lower mentorship dose due to higher numbers of facilities for the single mentor than in SK, the improvement seen in Kirehe was evidence that ABC could still be associated with meaningful improvement in different contexts.

For immediate skin-to-skin after birth, the baselines were reportedly starkly different between districts. Although this indicator existed in the HMIS before ABC, the register data source was not consistently available at baseline in Kirehe District, which may have contributed to the lower documentation of the practice at the start.

Management of preterm labor with antenatal corticosteroids was a new initiative and began from a low baseline in both district hospitals. Much of the initial work focused on district leadership advocacy to enable dexamethasone procurement by health centers, combined with clinical mentorship to align practice with emerging evidence on safety of steroid administration.^26^ Therefore, mentors guided clinical providers to provide steroids only when gestational age was known by last menstrual period, which was consistent with national protocol.

Surprisingly, we saw a significant increase in stillbirths in SK in the mixed-effects model. It is possible that despite improvements in target indicators related to improved labor management, stillbirths increased in the face of reduced neonatal deaths if access to quality postnatal care and complications management drove mortality reduction, and quality gaps in antenatal or labor management not captured in our performance measures remained. This possibility is the subject of further investigation.

Despite improved MNH quality of care and neonatal mortality, we found limited change in patient satisfaction. Mutaganzwa et al.^19^ described the baseline results in detail and found that patient-centeredness of care (including interpersonal relationships, respect, and privacy) and organizational factors such as cleanliness, comfort, and equipment/commodity availability were associated with higher satisfaction with care. Similar to other studies, one explanation for the lack of increased satisfaction corresponding with improved quality of care and outcomes could be that women were reluctant to share negative views or had low expectations of facility care and were satisfied with poor quality at baseline.^27^ It is also possible that the intervention did not have adequate focus directly on improving dignity and respect during patient contact care in the intervention, indicating that future programs may need intentional focus on patient-centered care as part of holistic quality to improve satisfaction.

Finally, despite the district-level variation in process level impacts, we found reductions in hospital case fatality and district neonatal mortality in the intervention area. The highest impact was seen among preterm/LBW infants, which has been a difficult area for improvement globally.^7^^,^^28^^,^^29^ The district-wide approach included supporting specialized care at the district hospital, which likely facilitated this finding and perhaps distinguishing the current study from other studies.^7^^,^^30^ Importantly, Rwanda has experienced health sector improvements over the past decade^8^^,^^31^ and would be expected to see some reduction in neonatal mortality; however, analysis of available data from the rest of rural Rwanda did not find a similar reduction.

Hospital case fatality and district neonatal mortality decreased in the intervention area, with the highest impact occurring among preterm/LBW infants.

The ABC approach was designed in collaboration with the MOH national and district leadership to enhance scalability and sustainability, which led to the integration of the improvement collaborative components into existing district structures. Based on the results of this program, MOH has continued scaling the approach into non-PIH-supported districts with funding from a Saving Lives at Birth award, with an impact evaluation underway. Furthermore, the program design worked to increase sustainability by building capability of health system actors at multiple levels—district leadership to oversee the approach and integration into routine district systems, and district supervisors to incorporate the mentorship and improvement methodology into their routine work. A sustainability study has been completed to understand system performance 1 year after the end of the intensive period and results will be published separately. As with all health system interventions, integration of effective approaches into routine administrative budgets can be a challenge. Demonstration of the technical impact and program ownership by local leaders were important to support incorporation of the core components (supervisor transport, review meeting costs, clinical and QI continuous learning) into district budgets, and they are ongoing efforts in financially constrained systems.

### Limitations

Our study had some limitations. Using HMIS and programmatic data meant that we were limited by the quality of the data available. We accounted for this by register review for all possible measures; however, this step would not address issues of accuracy or availability of paper registers. Facilities needed variable lead time to achieve consistent documentation of newly introduced indicators in preterm delivery management, and such documentation was sometimes incomplete. That said, given the growing global call to measure preterm labor management indicators, we hope this practical experience will have global relevance.^32^

In addition, mentors coached QI teams to document change ideas and proximal process data rigorously to determine “success” of a given change. However, we cannot be certain that all successful changes were included in the published change package to fully explain the causal pathway to measured improvement in quality of care and mortality.^16^ Some other time-intensive activities were difficult to capture in program documentation. These included documenting the specific focus of clinical mentorship activities at facilities and the work targeting coordination and communication within and across facilities to strengthen complications management.

Importantly, we did not have a comparison area with measurement and register comparison of mortality; therefore, we used the national HMIS data for rural districts. It is also possible that unknown differences were present between the intervention area and the rest of rural Rwanda. The intervention baseline mortality appears starkly higher than the mortality in the comparison districts, which could be easier to reduce. However, sensitivity analyses using HMIS routinely reported data from the intervention area showed a more comparable baseline mortality, and based on 2010 DHS analyses, the neonatal mortality rates in SK and Kirehe were similar to the rest of rural Rwanda.^12^ We believe the baseline mortality reported in the comparison districts to be low at least in part due to consistent underreporting in the HMIS, which was overcome in the intervention districts by extracting data from the register records.

Furthermore, improving data quality was part of the QI process, so the difference in differences results could be confounded by change in data quality over time.^33^ Routine review of HMIS mortality data compared with registers demonstrated underreporting of poor outcomes at baseline, which decreased throughout the intervention period. If a similar improvement in data quality occurred in the comparison area, it could have reduced the effect size. However, to our knowledge, no concurrent major data quality initiatives were occurring elsewhere in the country.

Mentors triangulated data across registers to use the highest quality data possible for QI and to improve the HMIS data quality. For example, birth weight and gestational age were recorded in the birth register. Mentors compared these data with the counts listed in the HMIS report tally for the same data elements to provide feedback on HMIS data quality to the facility data officer and to build awareness and data quality capability among facility staff in the process. HMIS data QI would not influence the reported quality measures; however, if reporting in the paper registers improved, it would likely lead to underestimation of effect size because it would bias results towards the null in most cases.

Finally, with the introduction of 3 new maternity units in Kirehe District, it is possible that the opening of the 3 new health centers has allowed a decrease of number of patients seeking care in other HCs, which could have contributed to improve the quality of care in the facilities surrounding them by allowing more time per patient; however, we did not track the differences in patient flow.

## CONCLUSION

We found that the ABC initiative was a feasible and effective program to improve MNH quality of care and reduce neonatal mortality in the intervention districts after 18 months. The QI approach enabled joint problem solving across program and MOH leadership when challenges were encountered. Full transition and further evaluation of sustainability is underway and the Rwanda MOH is currently scaling up ABC into additional non-PIH-supported districts. A mixed-method sustainability analysis will be reported elsewhere. To our knowledge, this study is the first to trace a multilevel neonatal QI program from implementation to clinical process, to mortality impact.^24^ As countries strive to achieve quality universal health coverage, the ABC initiative could be an important tool for leaders and implementers in countries looking to improve quality and reduce neonatal mortality.

## Supplementary Material

21-00031-Tibbels-Supplement.pdf

## Notes

**Peer Reviewed**
