# Supplementary material for: The All Babies Count Initiative: Impact of a Health System Improvement Approach on Neonatal Care and Outcomes in Rwanda
Source: Glob Health Sci Pract. 2020 Sep 30;8(3):0. doi: 10.9745/GHSP-D-20-00031 (PMC7541121; doi:10.9745/GHSP-D-20-00031)
Supplement: 21-00031-Tibbels-Supplement.pdf [file 21-00031-Tibbels-Supplement.pdf]

2

REAL-TIME RUMOR TRACKING FOR COVID-19

System Design and Implementation Guide

USAID
FROM THE AMERICAN PEOPLE

Breakthrough ACTION
FOR SOCIAL & BEHAVIOR CHANGE

Table of Contents

Acronyms **2**

Acknowledgements **3**

Overview **4**

COVID-19 and the Role of Rumors 4

The Purpose of Tracking Rumors 4

The RT² Approach **5**

Implementation 5

Component A: Build the Software Platform 5

Component B: Recruit and Train a Network of Community-Based Correspondents 5

Component C: Data Managers Record and Code Rumors and Display Them in Real Time 6

Component D: Those Working in RCCE Use Rumor Data to Inform the COVID-19 Response 6

Ethical Considerations 7

The DHIS2 RT² Metadata Package **8**

Overview of Metadata 8

Data Structure 8

Installation 9

Data Capture 9

Monthly Rumor 14

Other Metadata Elements 15

User Groups 15

User Roles 15

Program Rule Variables and Program Rules 15

Program Indicators 16

Program Indicator Name 16

Dashboard 17

Editing the Rumor Log 17

References **27**

Acronyms

API	Application programming interface
CBC	Community-based correspondent
CHW	Community health worker
DHIS2	District Health Information System 2
HMIS	Health Management Information System
NGO	Non-governmental organization
PRV	Program Rule Variables
RCCE	Risk communication and community engagement
RT^2	Real-time rumor tracking
UI	User interface

Acknowledgements

We acknowledge the many organizations who have come before in developing rumor tracking and community feedback systems, particularly approaches developed and implemented by the International Federation of Red Cross and Red Crescent Societies (IFRC) and by Internews. The Breakthrough ACTION team in Côte d'Ivoire, led by Johns Hopkins Center for Communication Programs, and the Centre for Communication Impact in South Africa contributed substantially in piloting the Breakthrough ACTION Real-time Rumor Tracking^2 approach. Development and piloting of the RT^2 approach and DHIS2 metadata package was made possible by the generous support of the American people through the United States Agency for International Development (USAID). The contents are the responsibility of Breakthrough ACTION and do not necessarily reflect the views of USAID or the United States Government.

Overview

This document is written for humanitarian or public health organizations as well as national governments seeking to document rumors in a systematic and dynamic fashion. First, the document provides an overview of the role of rumors in a public health or humanitarian emergency, followed by a summary of the community-based approach taken by Breakthrough ACTION during the COVID-19 pandemic. Finally, the guide lays out an application of this approach using the District Health Information System 2 (DHIS2) open source software platform, including an overview of a pre-configured metadata package that can be installed on a new DHIS2 system or imported to an existing system. While this guide and the associated DHIS2 package were developed for COVID-19, rumors play a role in a variety of public health issues. Real-time monitoring of rumors thus provides a unique opportunity for dynamic two-way communication with communities.

COVID-19 and the Role of Rumors

In January 2020, the novel coronavirus SARS-CoV-2 was identified as the cause of a multi-system illness now known as COVID-19. From December 2019 to the present, the virus has spread globally, resulting in over 26 million cases and almost 900,000 deaths.[a] COVID-19 is, at the time of this writing, a pandemic and an international public health emergency.

The uncertainty around the virus has led to the propagation of unverified pieces of information that spread rapidly through groups, called rumors. People may spread rumors in good faith, thinking they are helping others with the information (misinformation), or they may spread rumors with the intention to harm others or destabilize populations (disinformation).[b] The World Health Organization labelled the COVID-19 crisis an "infodemic" due to the plethora of misinformation circulating as well as the information overload for people seeking trustworthy guidance.[c] Rumors can undermine a robust public health response by reducing trust in health authorities, thus promoting harmful practices, creating barriers to protective practices, and even causing illness or death.[d] Risk communication and community engagement (RCCE) are critical to building trust and arresting the spread of rumors.[e]

The Purpose of Tracking Rumors

Rumor tracking is one way to improve RCCE during a pandemic. Real-time rumor tracking (RT^2) is the process of systematically collecting, analyzing, visualizing, and addressing COVID-19 rumors in real-time. Rumors can be identified through a range of sources including embedded community correspondents, national hotlines, or social media. This guide will focus on community-based correspondents (CBC), who are an existing cadre of non-governmental organizations (NGOs) or within the health system or traditional leadership system, and are already involved in their communities through volunteers or within the health system (such as community health workers (CHWs)). CBCs may include local radio hosts with call-in shows, community health workers, staff of non-governmental organizations who do community-based work, or community/traditional leaders. They are typically unpaid, yet still receive a brief training on recognizing and reporting rumors they hear in the course of their daily activities. CBCs can submit rumors directly to a cloud-based database, but more commonly, when time or data literacy is limited, they can submit the rumors to a WhatsApp line. The same WhatsApp connection also facilitates two-way communication, as factual information can be disseminated via the same connection.

---

[a] Center for Systems Science and Engineering at Johns Hopkins. (2020). COVID-19 Dashboard. Johns Hopkins University and Medicine Coronavirus Resource Center. https://coronavirus.jhu.edu/map.html

[b] Bugge, J. (2017). Rumour has it: A practice guide to working with rumours. CDAC Network. (A. Rouse, Ed.) http://www.cdacnetwork.org/tools-and-resources/i/20170613105104-5v7pb

[c] The Lancet Infectious Diseases. (2020). The COVID-19 infodemic. The Lancet Infectious Diseases, 20(8), 875. https://doi.org/10.1016/S1473-3099(20)30565-X

[d] Islam, M. S., Sarkar, T., Khan, S. H., et al. (2020). COVID-19-related infodemic and its impact on public health: A global social media analysis. The American Journal of Tropical Medicine and Hygiene, https://doi.org/10.4269/ajtmh.20-0812

[e] Breakthrough ACTION. (2020). COVID-19 Rumor Tracking: A Technical Brief for Breakthrough ACTION Field Teams. Breakthrough ACTION and RESEARCH. https://breakthroughactionandresearch.org/wp-content/uploads/2020/05/COVID-19-Rumor-Tracking-Technical-Brief_v1.1.pdf

The RT² Approach

This document describes the approach to community-based real-time monitoring of rumors, then describes the system design for tracking rumors using DHIS2 open source software. DHIS2 is a secure web server and data collection, storage, management, and analysis platform used in over 60 countries, including by several ministries of health for reporting, analysis, and visualization of surveillance data. The approach can also be implemented using other software platforms by configuring the rumor log in ODK, Excel, or other platforms. However, this metadata package with DHIS2 offers a streamlined approach to collection, storage, and visualization.

Tracking rumors in real-time through community-based liaisons allows governments and those working in RCCE to identify and respond rapidly to actionable rumors.

Implementation

The DHIS2 system is only one of several components of the real-time rumor tracking approach. The whole approach includes:

1. The software platform
2. The network of community-based liaisons who listen to communities and submit rumors immediately
3. The data managers who process and code the data
4. Those working in RCCE who interpret and use the rumor data to inform the pandemic response

There are several steps to implementing this comprehensive approach:

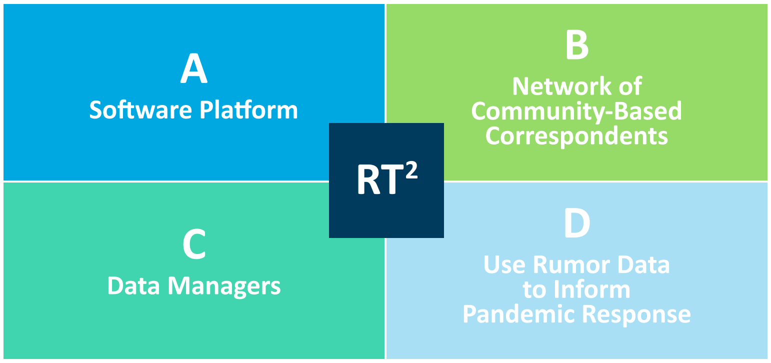

Component A: Build the Software Platform

A database with real-time visualization capabilities elevates rumor management by making rumor data available and understandable to responders. There are a variety of software platforms that can work, and implementers must weigh simplicity against desired features. A shared Google sheet may be sufficient in some cases, whereas in other cases, the need to manage a large volume of rumor submissions or the desire to visualize the data using continually updated maps or charts would necessitate a platform like DHIS2.

Component B: Recruit and Train a Network of Community-Based Correspondents

Community-based correspondents receive virtual or face-to-face training on the importance of tracking rumors, how to recognize a reportable rumor and how to submit rumors. CBCs are instructed to submit the rumor each time they hear it, even if it is a duplicate, to give a sense of how widespread and in which geographic areas similar rumors may be circulating. CBCs are motivated by the key role they play in the country's COVID-19 response, a reasonable request if their CBC role does not add additional work apart from submitting the WhatsApp or email message. The advantage of having volunteers and CHWs as CBCs is that their function as correspondents builds on an *existing and trusted community-embedded network* rather than hiring new staff whose only community engagement role is rumor tracking.

In the course of their regular work, CBCs will hear rumors related to COVID-19. Rumors may be shared unprompted during interactions with community members. If possible, existing monitoring forms, regularly used by these CHW and volunteers, can include a new field, "What have you heard recently about COVID-19 that you're not sure is true?" CBCs submit the rumor to a designated WhatsApp or email address. As CBCs interact with community members and hear rumors related to COVID-19, they submit them to a central email or WhatsApp line in an unstructured, qualitative form (a couple of sentences summarizing the rumor, stating it as they heard it from the source). In some cases, countries may want the CBCs to code the rumors, but typically data managers code more consistently and it reduces the barrier to submitting rumors for CBCs if others do the coding, and limiting their work to accurate and timely reporting.

Component C: Data Managers Record and Code Rumors and Display Them in Real Time

Data managers record and code the rumors received from CBCs in a database using a structured questionnaire and codebook (rumor log). Rumors from other sources such as social media can also be incorporated using the same log. Topical codes are already included in the log and were developed based on a review of the literature and experiences in countries piloting the system. Topical codes fall into one of the following categories:

- I **Knowledge and Attitudes:** Beliefs about the populations at risk (who is susceptible or not), suspect cases or deaths, denial of the illness, status/end of the epidemic, origin of the illness, fear/perceived severity of the illness, and conspiracy theories
- II **Sources/Transmission of COVID-19:** Mode of transmission, hygiene
- III **Signs and Symptoms:** Any signs or symptoms believed to be associated with COVID-19
- IV **Prevention and Treatment of COVID-19:** Vaccination, prevention, treatment/cure, traditional or religious medicine, seeking care at health facilities for testing or treatment
- V **Government Response and Pandemic Outcomes:** Confinement, quarantine, or distancing, estimates of case counts or deaths, access to resources (masks, medicines, finances), government response, consequences of the pandemic, gender, stigma
- VI **Other (Specify):** Data managers are given the opportunity to suggest new codes that may not have been previously captured.

Data managers should refer rumors that require an immediate response to the necessary focal points (for example, to medical, surveillance, or legal experts). A field in the form can be used to indicate whether an immediate response is necessary based on the nature of the rumor.

Rumor data is displayed in real-time on custom dashboards. We organized rumors according to constructs of behavioral theories using visualizations to facilitate risk communication decision-making. Data managers also summarize the results at pre-defined frequency based on the emergency status.

At periodic intervals, data managers in collaboration with those working in RCCE examine the coded raw data as well as the dashboard visuals and extract emergent themes. These themes tend to be phrased as "belief statements" which can be countered through the dissemination of accurate information. The emergent beliefs are shared with communication leads and may be added to the rumor log for further real-time tracking. This deeper analysis is key to both understanding how communities perceive the virus and providing a platform to capture their questions and beliefs.

Example belief statements:

"Treating coronavirus at home is safer than going to a health facility."

"COVID-19 is not a serious illness; it is just like the flu."

Component D: Those Working in RCCE Use Rumor Data to Inform the COVID-19 Response

The dashboard functionality allows those working in RCCE to identify actionable rumors quickly and take measures to "nip them in the bud" through mass media as well as returning factual information to the CBCs to help disseminate.

Ethical Considerations

Whether or not formal ethical oversight is sought, guiding principles should include:

- **Respect for Persons:** No individual, identifiable information should be entered into the database. The purpose of the RT^2 approach is to reflect general beliefs and perceptions, not implicate individual people or function as a surveillance system. CBCs should be trained not to submit individual names or addresses and to report dangerous or illegal activities to the proper authorities. The participation of CBCs and community members is completely voluntary. CBCs should not push or coerce, but rather obtain information in a natural way as they interact with people. They should be honest about their role, responding when asked that they are listening to people's perceptions about COVID-19 so the public health response can clarify and correct any misinformation.
- **Beneficence:** The purpose of the RT^2 approach is to benefit the people of each country employing the system, by providing a mechanism for feeding questions and rumors to the authorities who can then respond with accurate and trustworthy information. CBCs and data managers should make every effort to avoid harming individuals. Wherever it is possible, CBCs should be kept informed of resources with credible and up-to-date information on health and social issues like COVID-19.
- **Justice:** CBCs should be trained to report rumors equitably, including the voices of vulnerable populations, and not "editing" the rumors for personal gain. They should also share accurate information equitably.

NOTE: The rumors reported are only indicative of perceptions in geographic areas in which they were collected. As this is not a survey, the data are not population representative, but are rather a snapshot of emerging beliefs at a point in time.

The DHIS2 RT² Metadata Package

The RT² package was configured in version 2.33 and should be upgraded periodically as new DHIS2 builds are released. The word "metadata" in the context of DHIS2 refers to the objects in the system that define the data itself, such as data elements, indicators, organization units, and option sets. The rumor log is configured as an event program, and the metadata package includes:

1. Relevant user roles and user groups
2. The event program (Rumor Log) and recommended data elements
3. Program indicators
4. Dashboards that summarize both aggregate and raw rumor data

Overview of Metadata

A DHIS2 system or installation is called an "instance." Two DHIS2 instances are not automatically linked simply by virtue of being DHIS2. However, linking data from multiple DHIS2 instances (either through an automatic integration or through manual exports and imports) is easier than going between DHIS2 and other platforms because the basic system architecture is the same. The metadata included in this RT² package can be installed on an existing DHIS2 instance, such as a Health Management Information System (HMIS) or a project Monitoring & Evaluation system, but it can also be installed on a new DHIS2 server. Configuring and maintaining DHIS2 can be performed, for the most part, either through the application programming interface (API) or the user interface (UI), which can be accessed through a web browser. DHIS2 UI takes an app-based approach. Apps can be accessed in the top right corner.

Data Structure

There are three essential dimensions that describe each piece of data in DHIS2: organizational units (where), data elements (what), and time period (when). These dimensions influence the design of the metadata package as follows:

- **Organizational Level:** Countries piloting the RT² approach have been collecting rumors at the district level (typically the second or third sub-national level), which allows for a tailored response without running the risk of identifying individuals.
- **Data Elements:** The data elements are the questions or fields in the rumor log and describe what is asked for or counted in the form, such as, "Source of the rumor" or, "Topic: Access to resources." Data elements can have different value types, such as "Yes Only" (a check box), "Yes/No," "Text," "Integer," or become available via a dropdown menu (called an option set). Option sets for the relevant data elements are included. Program indicators are also included in the package. Program indicators are calculations based on the raw data elements that help you query the data with more flexibility.
- **Period:** The period dimension defines when the data were collected, such as when the rumor was received by the data management team and entered into the database. We have chosen an event program for the rumor log to enable rumors to be captured as close to the date and time they are received as possible.

Installation

In order to set up a new DHIS2 server, you will need to follow the instructions in the DHIS2 server configuration guide. You may also prefer to hire a vendor to handle your hosting and configuration.

After you have created the server and added your organization units, or if you have an existing DHIS2 instance, you can then install the JSON files for the RT2 package. Navigate to the “Import/Export” application in DHIS2, then choose “Metadata import.” Install each of the JSON files in order, starting with user groups. Clear your cache before proceeding.

Data Capture

Upon login, navigate to the Capture app.

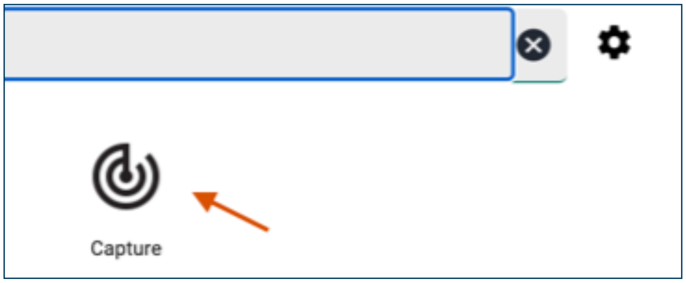

Figure 1

Choose the proper operational unit and select the Rumor Log program. Click “New Event”.

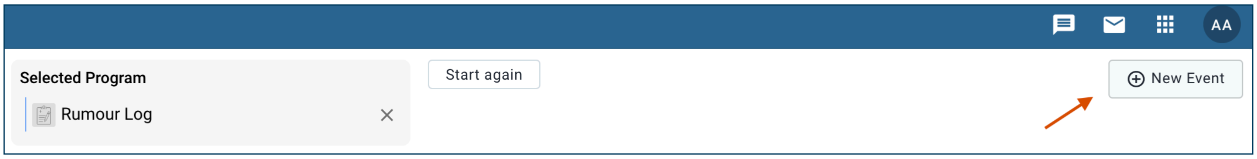

Figure 2

The form will appear. You will enter characteristics, such as the date the rumor was received by the data management team, the code of the person entering the data, the source of the rumor, and the message itself. Enter the submission verbatim as received from the community.

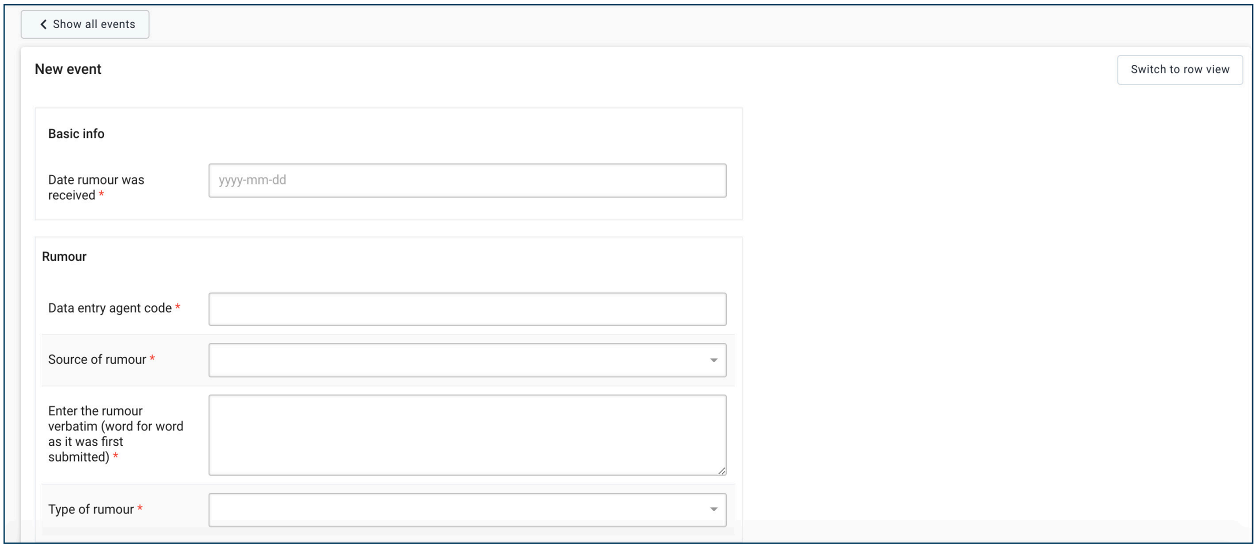

Figure 3

Data managers “code” each submission, which simply means they tag it according to certain topics and belief statements. This allows the rumors to be easily counted and organized for RCCE purposes. Answer each question and check the box for each relevant topic or belief. The following table lists each data element and how to code the rumor.

CODE	DESCRIPTION
**Source of rumor**	Describe who submitted the rumor or where the rumor was found. Do not try to trace it back to the source. If the rumor was mentioned by multiple sources, enter it multiple times.
**Community correspondent code**	Enter the code of the community reporter who submitted the rumor.
**Data entry agent code**	Enter your own code (the code of the person entering and coding the data).
**Rumor**	Enter the rumor verbatim (word for word as it was first submitted).
**Type of rumor**	Check the box that best describes the type of rumor.
**Access to resources (masks, medicines, finances)**	Use this code if the rumor relates to individual, community, or national ability to access resources needed for COVID-19 prevention, treatment, or other resources as a result of the pandemic.
**Perceptions of who is at risk**	Use this code if the rumor relates to whether certain populations can become infected, or comparisons in the level of risk between different types of people. It may include age, ethnicity, gender, socioeconomic status, pre-existing conditions, or other factors.
**Characteristics of the virus**	Use this code if the rumor relates to how the virus behaves (e.g., mutations and contagiousness).
**Confinement, quarantine, or lockdowns**	Use this code if the rumor relates to lockdowns or quarantine procedures.
**Consequences of the pandemic**	Use this code if the rumor relates to the impact of the pandemic on economic or social aspects of daily life or communities, or other consequences of the pandemic.
**Conspiracy theory**	Use this code if the rumor describes a conspiracy theory (a theory that rejects official accounts of an event or situation in favor of secret organizations or secret plots).
**Contact tracing**	Use this code if the rumor relates to the process of following up contacts of known cases.
**Denial of the illness**	Use this code if the rumor relates to whether or not coronavirus exists.

CODE	DESCRIPTION
**Estimates of case counts or deaths**	Use this code if the rumor relates either to perceptions of the accuracy of published case counts or deaths or to feelings about the number of cases or deaths.
**Fear/perceived severity of the illness**	Use this code if the rumor relates to how serious the illness is or a sense of fear or foreboding about the pandemic.
**Gender**	Use this code if the rumor relates to gender norms or the ways in which experiences may vary by gender during the pandemic.
**Government response**	Use this code if the rumor relates to the government response, including perceptions of the success of the response or specific feedback or assessment of motivations.
**Mode of transmission**	Use this code if the rumor relates to how the virus is transmitted between people.
**Origin of the illness**	Use this code if the rumor relates to where the illness came from (e.g., animals or a laboratory).
**Prevention**	Use this code if the rumor relates to prevention methods such as wearing masks, social distancing, reducing large gatherings, hand washing, or theories about other foods, drinks, or processes that may prevent COVID-19.
**Seeking care for testing or treatment**	Use this code if the rumor relates to the process of obtaining healthcare at either general health facilities or COVID-19 testing or treatment centers.
**Self-efficacy**	Use this code if the rumor reflects whether individuals believe they are able to perform recommended prevention behaviors.
**Signs and symptoms**	Use this code if the rumor relates to the signs and symptoms of COVID-19.
**Status/end of the epidemic**	Use this code if the rumor either relates to the progress of the pandemic (e.g., infections or waves) or predicts the end of the pandemic.
**Stigma**	Use this code if the rumor either exhibits or describes stigmatizing attitudes or behaviors toward people who are infected, COVID-19 survivors, health workers, and particular ethnic or racial groups (including both beliefs and discriminatory treatment).
**Suspect case or death**	Use this code if the rumor relates to an actual suspected COVID-19 case or death. Do not enter names or locations of individuals, however; refer the rumor to surveillance experts if warranted.
**Traditional or religious medicine**	Use this code if the rumor describes theories about traditional or religious medicine as prevention or treatment for COVID-19, or if the rumor describes the use of traditional or religious medicine in communities.

CODE	DESCRIPTION
**Treatment/cure**	Use this code if the rumor relates to possible or actual treatments or cures for COVID-19.
**Vaccination**	Use the code if the rumor relates to vaccinations, either for COVID-19 or routine vaccinations.
**Other**	Use this code if you would like to propose a new theme that is not captured above.
**Other (specify)**	In a few words, describe the new theme.
**Flag for immediate response**	Use this code only if the rumor may warrant immediate action from law enforcement or surveillance experts.
**Rumor analyzed**	Use this code to document that the rumor has been coded and analyzed for beliefs and recommendations.
**Coronavirus vaccine tested w/o knowledge/ assent**	Use this code if the rumor reflects the belief that a vaccine against SARS-CoV-2 may be tested on an unwilling population (e.g., forcibly injected, required by the government, or disguised as routine vaccinations).
**Case counts over/ underestimated for political reasons**	Use this code if the rumor reflects the belief that official case counts cannot be trusted, that health officials are overestimating them for financial gain or underestimating them for political reasons, or that health workers are falsely diagnosing clients.
**Certain foods/drinks can prevent or cure**	Use this code if the rumor reflects the belief that either eating certain foods or drinking alcohol, hot water, or other beverages can prevent or treat coronavirus.
**Certain medications can prevent or cure**	Use this code if the rumor reflects the belief that medications, such as either malaria medications and antiretrovirals or other medications, whether informal or approved, can prevent or cure coronavirus.
**Coronavirus does not exist**	Use this code if the rumor reflects the belief that coronavirus does not exist or is an invention.
**Coronavirus only affects certain people**	Use this code if the rumor reflects the belief that not everyone can contract coronavirus.
**Recommended measures do not work or are harmful**	Use this code if the rumor reflects skepticism about whether recommended prevention measures work to prevent the spread of COVID-19, or suspicions that the measures are actually harmful or spread COVID-19.
**Self-medication or treating at home is better**	Use this code if the rumor reflects a preference for self-medication or home remedies for COVID-19.
**Reflections and recommendations**	Use this code if the rumor reflects a preference for self-medication or home remedies for COVID-19.

Once you have fully coded the rumor, you can click "Complete", then save and exit. If you plan to return to the rumor and code further, you can save an exit without completing the event.

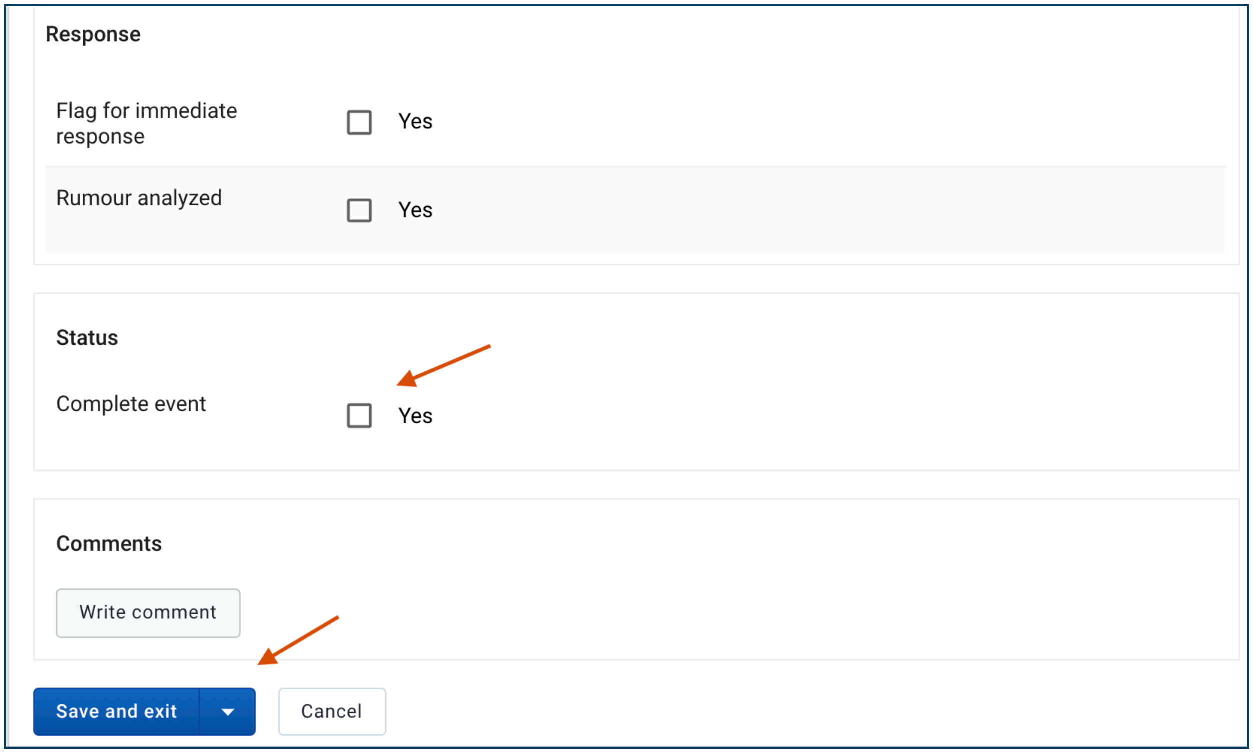

Figure 4

Monthly Rumor

In order to track how rumors have been addressed, there is a monthly aggregate form included at the sub-national level. The period and the level can be adjusted based on the reporting needs. The purpose of this form is to document how many rumors were addressed during the reporting period, and how they were addressed. To complete the form, navigate to the data entry app.

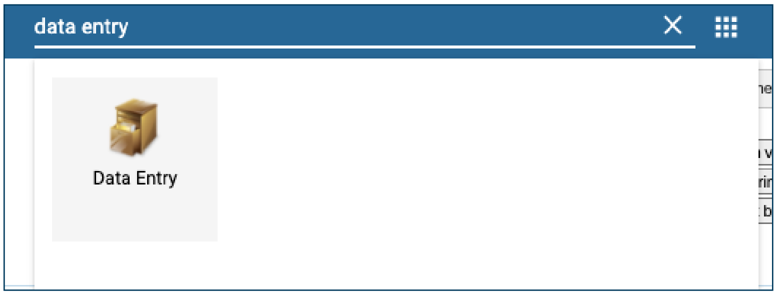

Figure 5

Select the organisation unit to which the data set has been assigned, and choose the correct reporting period. After the data are entered, click “complete”.

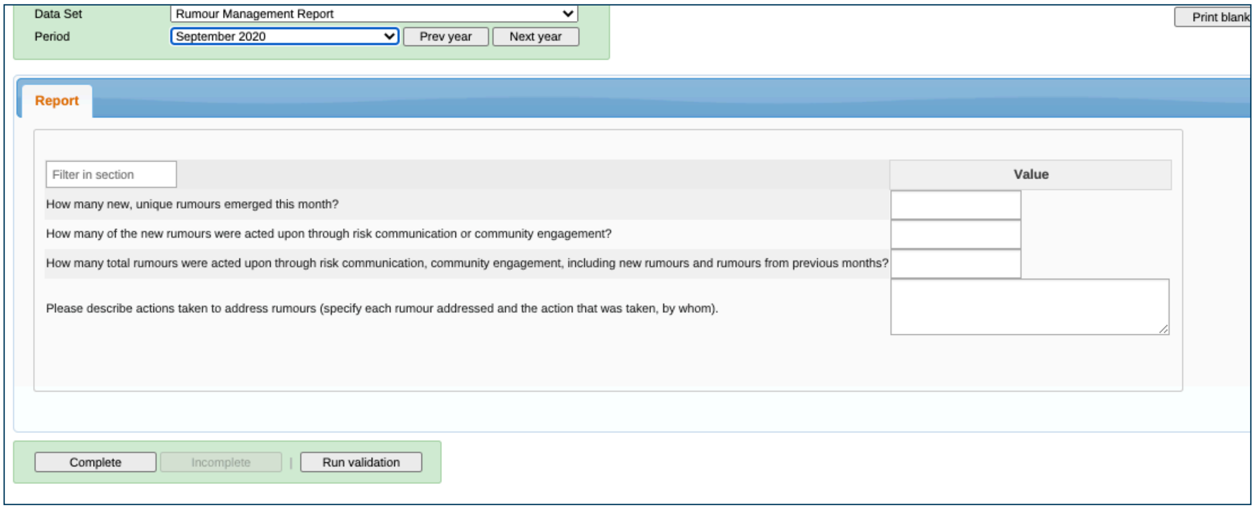

Figure 6

Other Metadata Elements

User Groups

The following user groups are recommended and are included in the metadata package:

- **Rumor data: Capture** (can enter data)
- **Rumor data: View** (can view data on dashboards or in analytics apps)

The rumor log event program and dashboards are currently shared with these two user groups with the appropriate permissions.

User Roles

The following user roles are recommended and are included in the metadata package:

- **Dashboard only:** Can only view the dashboard, without clicking into analytics apps
- **Analytics:** Can view dashboard and access all the analytics apps to create tables, graphs, and maps
- **Data entry:** Can enter rumors and code in the Rumor Log event program
- **Metadata edit:** Can edit the form and all metadata but cannot change scheduler jobs or perform maintenance tasks
- **Superuser:** All permissions

Program Rule Variables and Program Rules

The following program rule variables (PRV) are recommended and have been configured for the program.

PRV NAME	DESCRIPTION
**Other theme**	Data element in the current event
**Rumor analyzed**	Data element in the current event
**Source of rumor**	Data element in the current event

The following program rules are recommended and have been configured for the program.

PROGRAM RULE NAME	DESCRIPTION
**Show field "Community correspondent code" if source is community correspondent**	If the source of the rumor is a community-based correspondent, the form will ask for the code
**Show field "other (specify)" if "other" theme is selected**	If "other theme" is selected, the form will allow the data manager to describe the theme using free text
**Show section "Belief coding" if rumor analyzed is selected**	If the data element "RL-Response: Rumor analyzed" is selected, the section to code belief statements will appear

Program Indicators
The 45 program indicators included in this metadata package correspond to the basic analyses required in the analytics apps using the pre-set form.

Program Indicator Name

Belief: A coronavirus vaccine is being tested without our knowledge or against our will
Belief: Case counts are over or underestimated for political reasons
Belief: Certain foods or drinks can prevent or cure coronavirus
Belief: Certain medications can prevent or cure coronavirus
Belief: Coronavirus does not exist
Belief: Coronavirus only affects certain people
Belief: Recommended measures (e.g., masks, distancing) do not work or are harmful
Belief: Self-medication or treating at home is better than going to a facility
Rumors analyzed
Rumors submitted by community correspondents
Rumors submitted by health workers
Rumors submitted by radio stations
Rumors submitted from other source
Rumors submitted from social media
Rumors that require immediate review
Rumors theme: Access to resources (e.g., masks, medicines, finances)
Rumors theme: Characteristics of the virus
Rumor theme: Confinement, quarantine, or lockdowns
Rumor theme: Consequences of the pandemic (e.g., economic, social)
Rumor theme: Conspiracy theory
Rumor theme: Contact tracing
Rumor theme: Denial of the illness
Rumor theme: Estimates of case counts or deaths
Rumor theme: Fear/perceived severity of the illness
Rumor theme: Gender
Rumor theme: Government response
Rumor theme: Mode of transmission
Rumor theme: Origin of the illness
Rumor theme: Other
Rumor theme: Perceptions of who is at risk
Rumor theme: Prevention
Rumor theme: Seeking care at health facilities for testing or treatment
Rumor theme: Signs and symptoms
Rumor theme: Status/end of the epidemic
Rumor theme: Stigma
Rumor theme: Suspect case or death
Rumor theme: Traditional or religious medicine
Rumor theme: Treatment or cure
Rumor theme: Vaccination
Rumor type: Belief/misinformation
Rumor type: Event or situation
Rumor type: Feedback or suggestion
Rumor type: Other
Rumor type: Question
Total rumor submissions

Dashboard

Two pre-set dashboards have been configured:

1. **COVID-19 Rumor Tracking:** overall summary of rumor submissions by location, type, and topic. This allows RCCE actors to see which types of rumors are spiking or emerging by date and location.
2. **COVID-19 Topics and Beliefs:** This dashboard includes tables of coded rumors for each topic and for each belief statement. This allows you to “zoom in” on the topic or belief of interest and see the raw data.

For further analysis, users can create new tables and graphs which then can be added to custom dashboards. Dashboards can be created for any audience and shared with any user with a login. Note that dashboards must be shared with user groups or specific users in order to be viewed, *even by other system administrators.* As of version 2.34, graphs and tables can be configured in the “Data Visualizer” app. If boundary layers are configured for your country, maps can be configured in the “Maps” app. Raw data can be viewed in the “Event Reports” app.

Users who can view specific favorites through the dashboard can share interpretations. Comments can be posted on the visualizations by clicking the word bubble and posting. Other users can view and reply. Note that interpretations may be more public than the visualizations themselves, so proprietary information should not be shared in the comments.

Users can view all objects on a dashboard according to a particular time period or geographic location by clicking “Add filter.” This will override, temporarily, the settings of each individual graph or table, and will show you the data according to the time period or geographic unit you select.

Editing the Rumor Log

There are three steps to adding questions or codes to the Rumor Log.

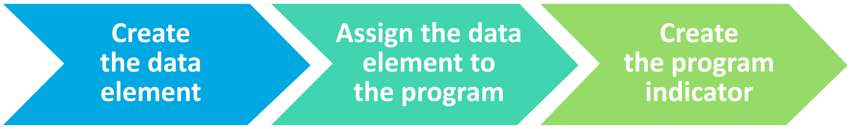

Step 1: Create the new data element.

First, go to the app “Maintenance” and click on the “Data element” box.

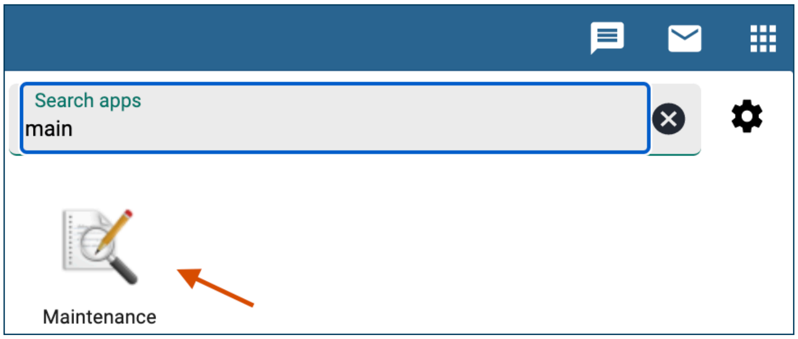

Figure 7a

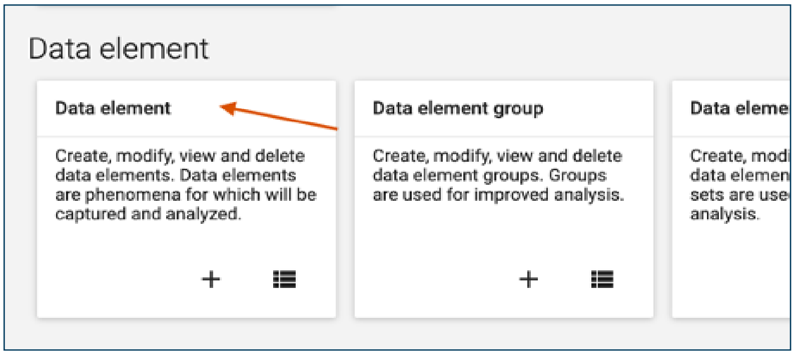

Figure 7b

Add a new data element to measure what you want to measure. You can click the blue plus sign to add a new data element from scratch.

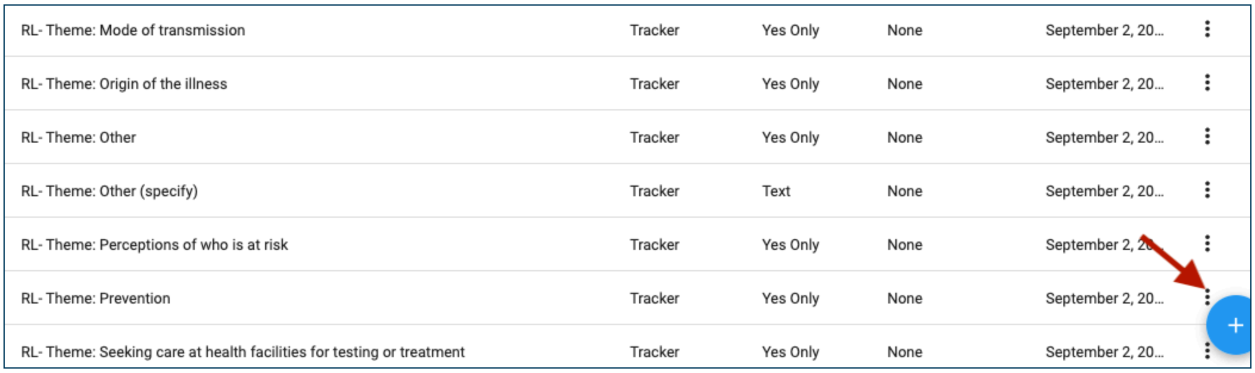

Figure 8

Alternatively, you can choose a similar data element using the three dots on the right-hand side, select “clone”, and rename it to reflect your new indicator.

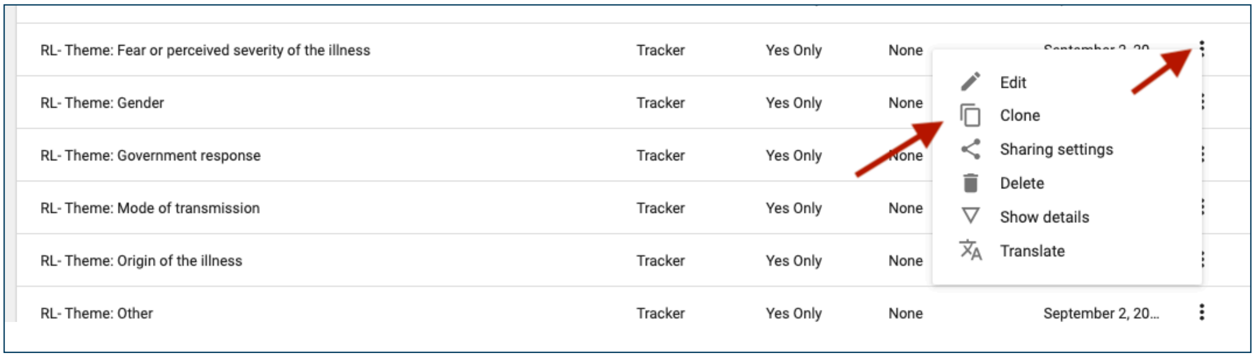

Figure 9

Give the data element a name (it can be any length and will be useful for organizing data elements into groups), a short name (limited to 50 characters; it displays in the analytics apps), choose the form name (what the user sees in the form), choose the domain type (“Tracker”), the value type (typically “Text,” “Yes Only,” “Yes/No,” or “Integer”), the aggregation type (typically “Sum”). The other fields can usually be left as the default. Click “Save.”

Step 2: Assign the data element to the Rumor Log

From the data element section, click over to the “Program” section and select the “Program” box.

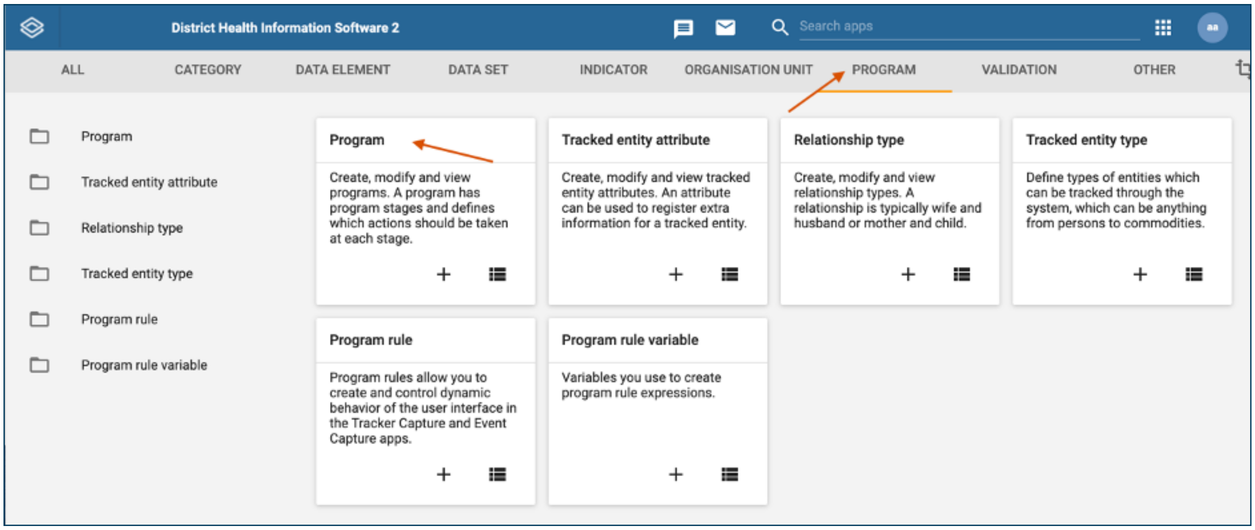

Figure 10

Click on the Rumor Log to open the form configuration screen.

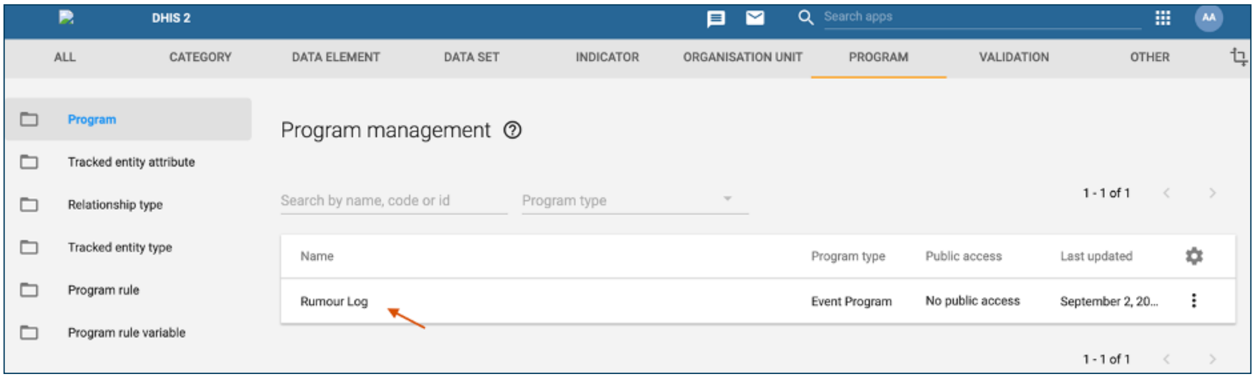

Figure 11

Move to the second tab, "Assign data elements." You will see the new data element(s) you just created. Double-click on any data element to move it over to the right side so it will be added to the form. If you created more than one and would like to move all newly created elements at once, you may select "Assign All" at the bottom of the page.

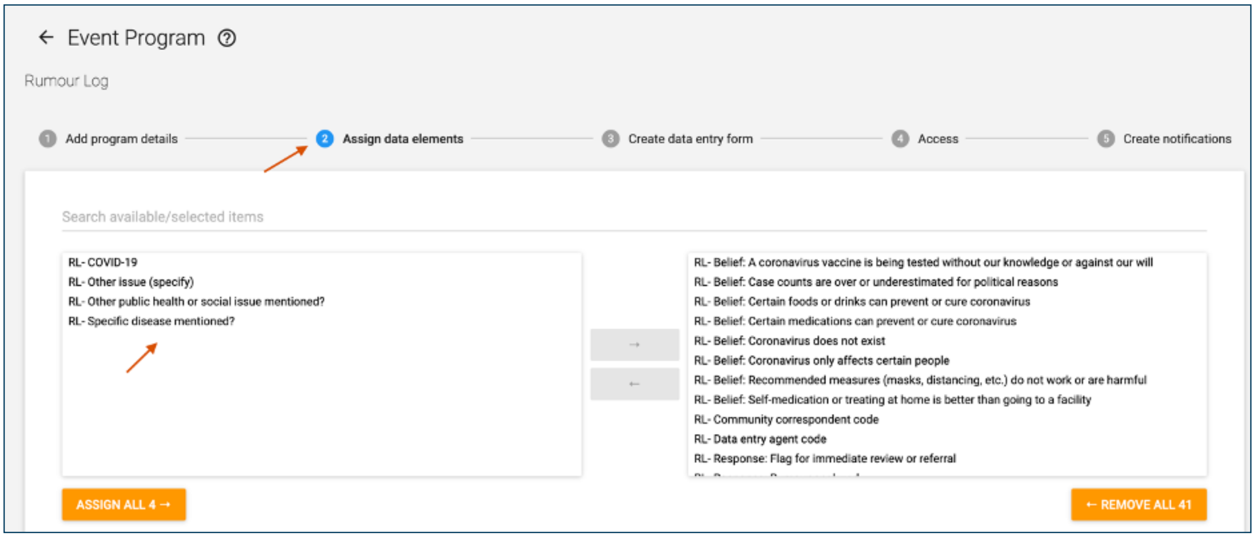

Figure 12

Move over to the tab, “Create data entry form.” The Rumor Log is a section form. Click on the section where the new data element should be added to ensure it is selected. Then click on the plus sign next to the new data element. Data elements that have already been added to the form are grayed out.

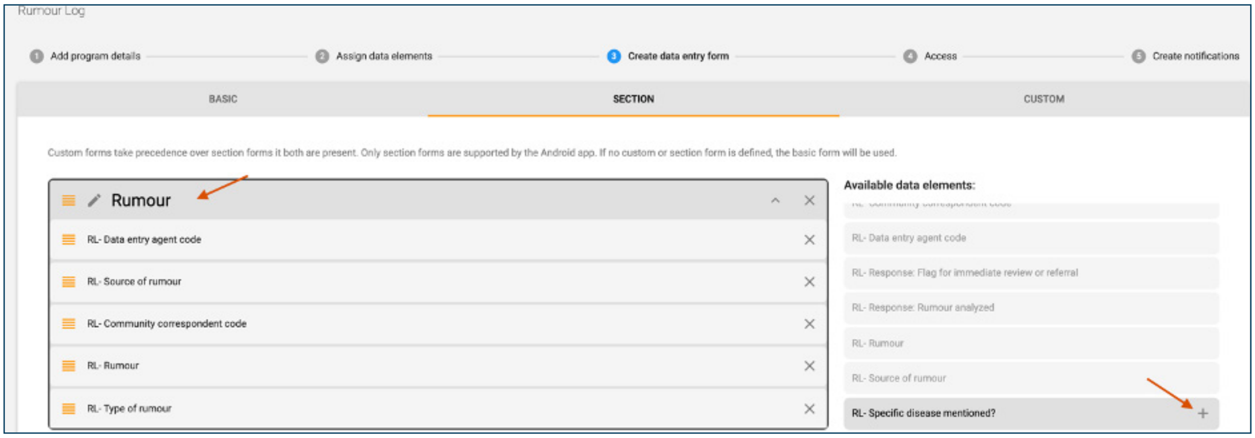

Figure 13

Drag the data element to change the order in the form.

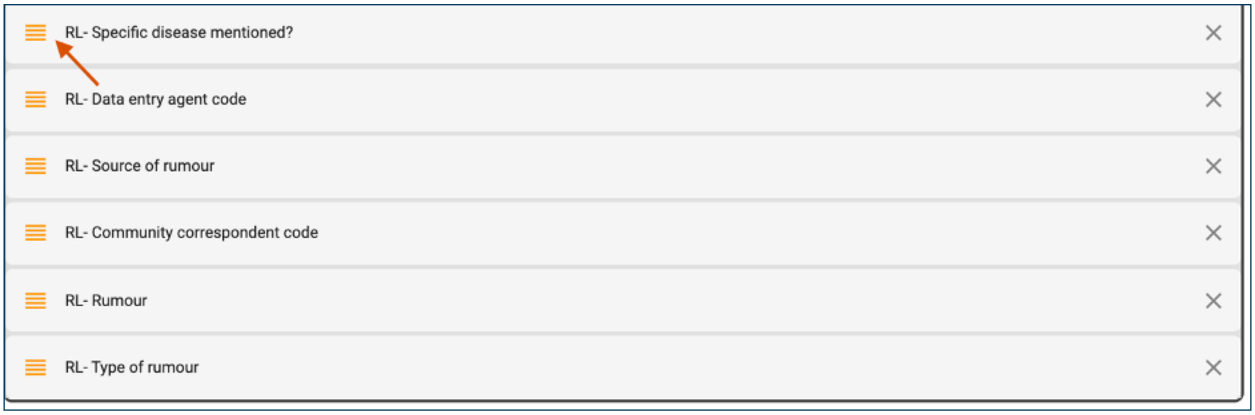

Figure 14

Navigate to the bottom of the page and click ”Save.”

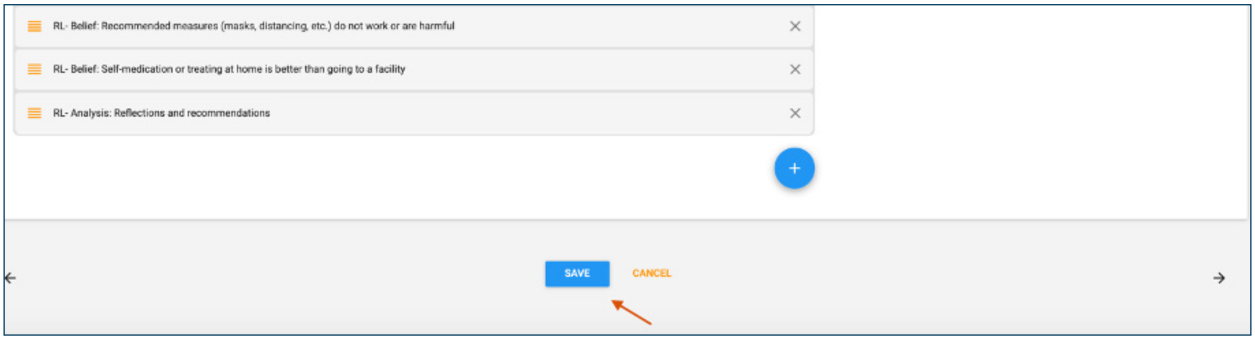

Figure 15

Step 3: Create a program indicator to count rumors based on the new data element

Now navigate over to the indicator section and select the “Program Indicator” box.

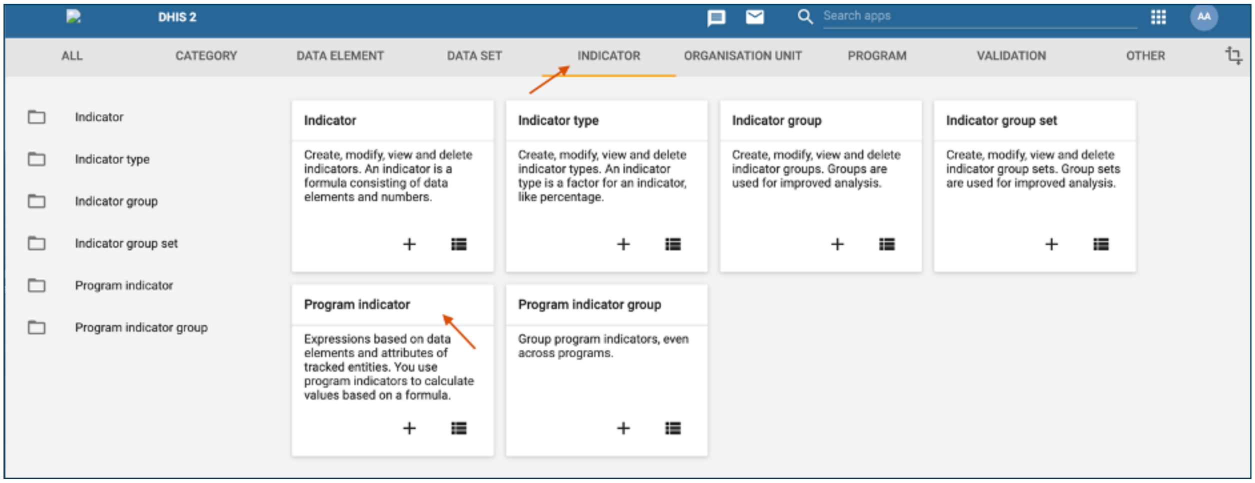

Figure 16

You can add a new program indicator or clone an existing indicator that is similar to what you have added.

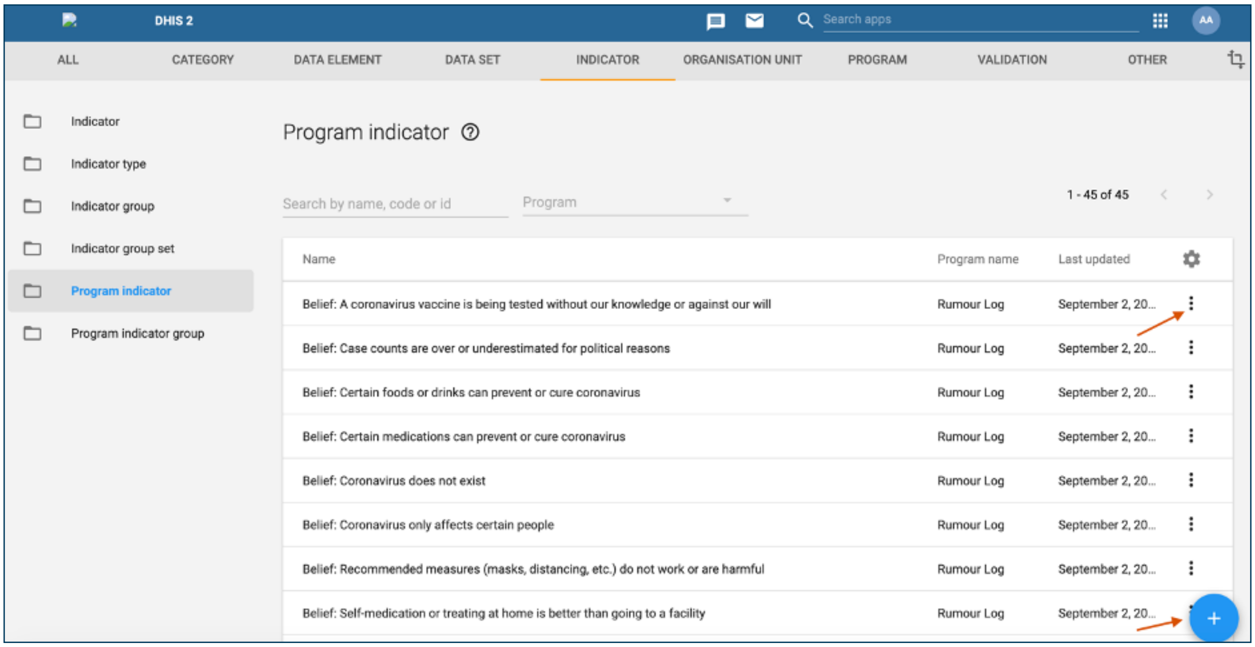

Figure 17

Select the program of interest under "Program (*)", and assign a name and short name to the indicator that reflects what you want the indicator to count.

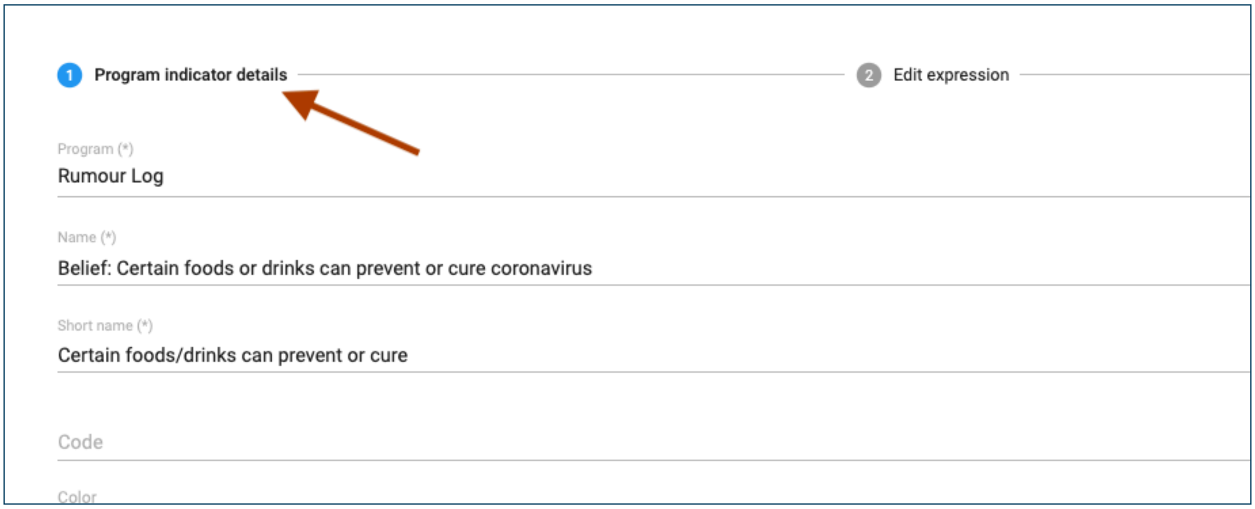

Figure 8

Typically, the aggregation type is "Count" and the analytics type is "Event."

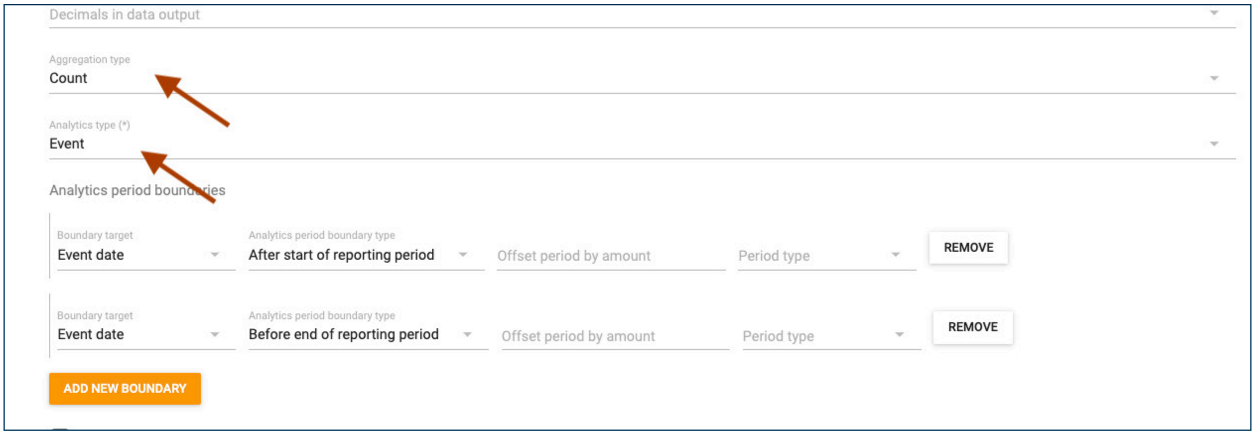

Figure 19

The expression is “Event count” which you can select from the list of “Variables” on the righthand side.

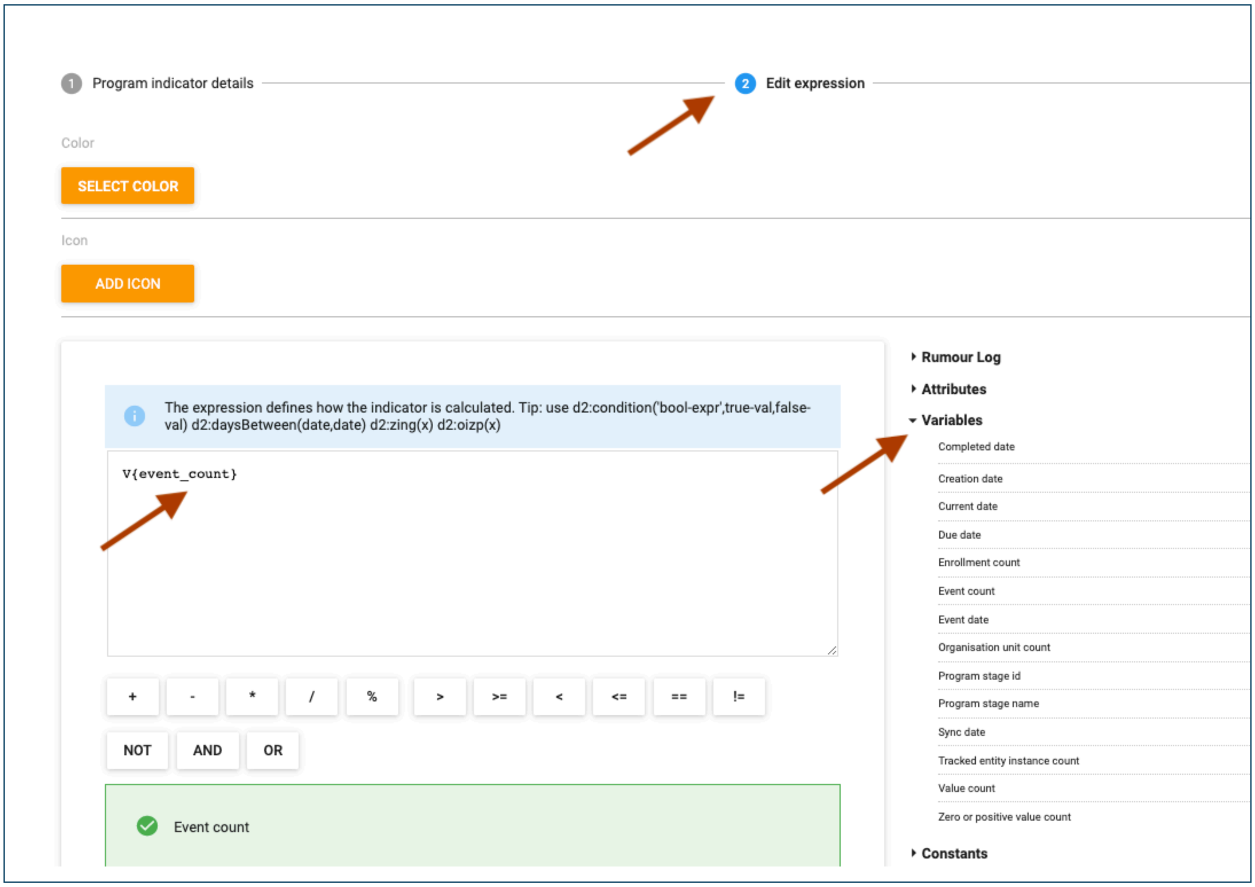

Figure 20

The expression tells the indicator how to filter the data. In this example, the expression tells the system to count all events (i.e., all rumor submissions). The filter instructs the system then to only count the rumor submissions where the response to this data element is “Yes” (a “Yes” value is “1” and a “No” value is “0” for the ”Yes/No” value type).

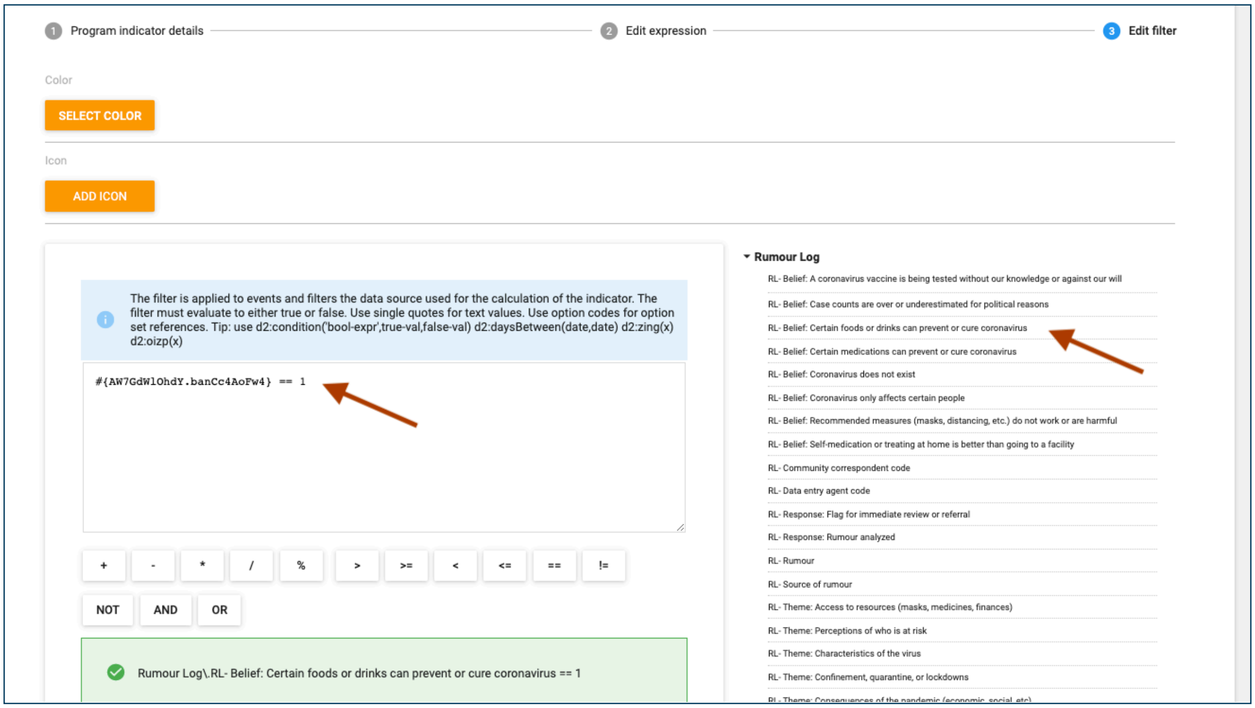

Figure 21

The filter will automatically show the unique code for that data element. You can verify the expression below the filter box.

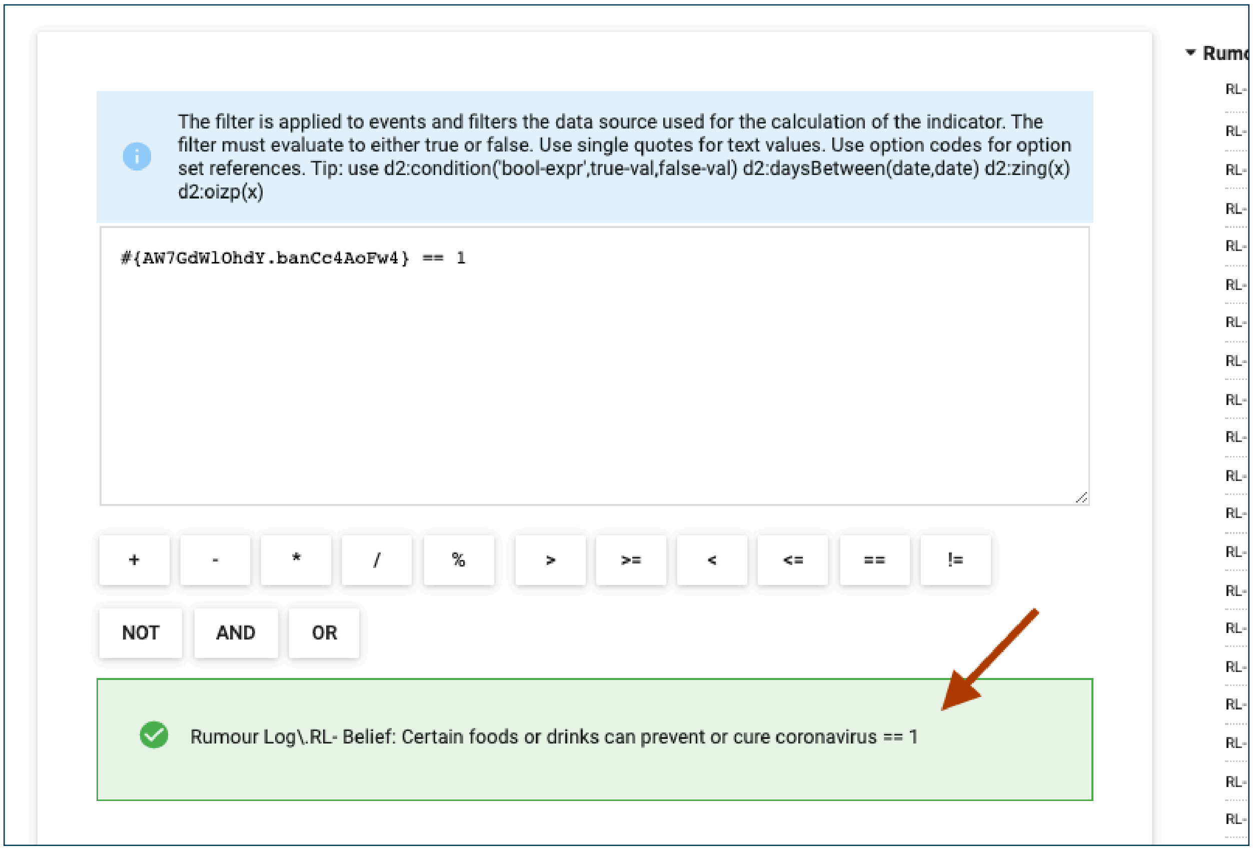

Figure 22

Click "Save," clear your cache, and you will see the program indicator in the analytics apps.

References

DHIS2 documentation: http://docs.dhis2.org

COVID-19 DHIS2 packages: https://www.dhis2.org/covid-19

COVID-19 Rumor Tracking: A Technical Brief for Breakthrough ACTION Field Teams: https://breakthroughactionandresearch.org/wp-content/uploads/2020/05/COVID-19-Rumor-Tracking-Technical-Brief_v1.1.pdf0
